# Redox Potential Tuning of s-Tetrazine by Substitution of Electron-Withdrawing/Donating Groups for Organic Electrode Materials

**DOI:** 10.3390/molecules26040894

**Published:** 2021-02-08

**Authors:** Dong Joo Min, Kyunam Lee, Hyunji Park, Ji Eon Kwon, Soo Young Park

**Affiliations:** 1 Lab for Supramolecular Optoelectronic Materials (LSOM), Department of Materials Science and Engineering, Research Institute of Advanced Materials (RIAM), Seoul National University, 1 Gwanak-ro, Gwanak-gu, Seoul 08826, Korea; mdj88@snu.ac.kr (D.J.M.); lgn0427@snu.ac.kr (K.L.); 2018-20150@snu.ac.kr (H.P.); 2 Functional Composite Materials Research Center, Institute of Advanced Composite Materials, Korea Institute of Science and Technology (KIST), 92 Chudong-ro, Bongdong-eup, Wanju-gun, Jeonbuk 55324, Korea

**Keywords:** s-tetrazine, organic electrode, Li ion battery, potential tuning

## Abstract

Herein, we tune the redox potential of 3,6-diphenyl-1,2,4,5-tetrazine (DPT) by introducing various electron-donating/withdrawing groups (methoxy, t-butyl, H, F, and trifluoromethyl) into its two peripheral benzene rings for use as electrode material in a Li-ion cell. By both the theoretical DFT calculations and the practical cyclic voltammetry (CV) measurements, it is shown that the redox potentials (E_1/2_) of the 1,2,4,5-tetrazines (s-tetrazines) have a strong correlation with the Hammett constant of the substituents. In Li-ion coin cells, the discharge voltages of the s-tetrazine electrodes are successfully tuned depending on the electron-donating/withdrawing capabilities of the substituents. Furthermore, it is found that the heterogeneous electron transfer rate (*k_0_*) of the s-tetrazine molecules and Li-ion diffusivity (*D_Li_*) in the s-tetrazine electrodes are much faster than conventional electrode active materials.

## 1. Introduction

Recently, interest in organic electrode materials has been growing rapidly due to the abundance and light weight of their elements, prospect of low cost, environmental friendliness, and structural diversity [1,2,3]. Moreover, in contrast to inorganic electrode materials based on metal oxides, the organic electrode materials can be applied to various types of metal-ion batteries such as Li-, Na-, K-, and divalent metal-ion batteries [4,5,6]. To date, an enormous number of organic electrode materials has been reported; however, they still mostly rely on only a few kinds of redox centers including quinone [7,8], imide [9,10], nitroxide [11,12], organosulfur [13,14], and carboxylate [15,16], which limits their practical performance inferior to their counterparts.

In this context, our group very recently proposed 1,2,4,5-tetrazine (s-tetrazine) as a new redox center for organic electrode materials for the first time [17]. The s-tetrazine redox center is promising for electrode-active material of metal-ion batteries because it can be reversibly reduced by one electron to form a stable radical anion due to its strong electron-deficiency [18,19]. Therefore, it is expected to theoretically deliver a high specific capacity as high as 327 mAh g^−1^ by virtue of its small molecular weight (M_w_ = 82 g mol^−1^). Furthermore, there is also a chance to greatly increase its theoretical specific capacity up to 654 mAh g^−1^ if the second one-electron reduction reaction of the s-tetrazine can be induced to reversibly occur by optimizing electrolytes and/or chemical structure modification [19,20,21].

In the previous study, we evaluated the applicability of s-tetrazine redox center for electrode materials using four simple s-tetrazine derivatives as model compounds. Among them, 3,6-diphenyl-1,2,4,5-tetrazine (DPT) showed a reversible one-electron redox reaction with an insertion/de-insertion of a Li-ion in a coin cell, which was evidenced by ex-situ XPS analysis [17]. It was ascribed to the fact that the two phenyl rings of the s-tetrazine core at 3- and 6-position prevented the unwanted side reactions. However, its discharge voltage was 2.24 V vs. Li/Li^+^, which lies in an intermediate voltage range between the typical cathode and anode. Therefore, redox potential tuning of s-tetrazine is highly desired for its practical use.

Fortunately, it is well known that the redox potential of organic materials can easily be tuned by the introduction of electron-donating or -withdrawing substituents [22,23,24,25]. For instance, Vadehra et al. [22] achieved 0.09 V and 0.54 V of elevation in the redox potential of naphthalene diimide (NDI)-based electrode materials by substitution of F and CN groups, respectively. Similarly, Kim et al. [23] reported that the discharge voltage of *p*-benzoquinone was elevated by 0.32 V through introducing four chlorine atoms. Park et al. [24] also showed that a disodium terephthalate (Na_2_TP) substituted by electron-donating amino-groups exhibited decreased redox potential in a Na-ion cell. Typically, the energy level of frontier molecular orbitals (FMOs) of organic molecules can be modulated depending on the electron-donating/withdrawing capability of the substituents, resulting in a redox potential change.

In this study, we introduced various substituents possessing different electron-withdrawing/donating capabilities into the s-tetrazine redox center to tune its redox potential systematically. To this end, a series of DPT derivatives bearing methoxy (MeO), t-butyl (t-Bu), H, F, and trifluoromethyl (CF_3_) groups was designed and synthesized. Using quantum chemical calculation methods based on density functional theory (DFT), the lowest unoccupied molecular orbital (LUMO) levels and reduction potentials of the s-tetrazines were theoretically calculated. Then, the electrochemical properties of the DPT derivatives including the redox potential (*E_1/2_*), the diffusion coefficient (*D*), and the heterogeneous electron transfer rate constant (*k_0_*) were evaluated by cyclic voltammetry (CV) measurements. Finally, the discharge voltages of the s-tetrazine electrodes measured by galvanostatic charge/discharge test was found to correlate well with the predicted redox potentials.

## 2. Result and Discussion

### 2.1. Materials Design and Synthesis

**Figure 1:**
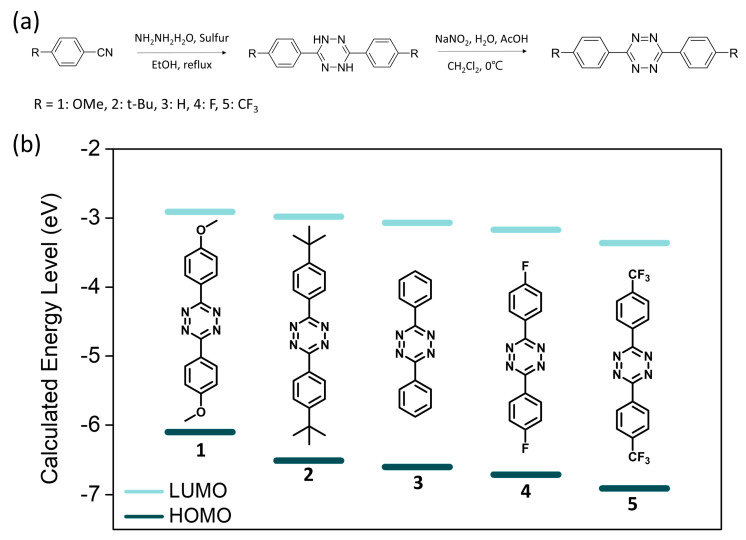
(**a**) The synthesis scheme of the s-tetrazine derivatives. (**b**) The calculated frontier molecular orbital (FMO) energy levels of the s-tetrazines calculated by DFT in solvated system.

As shown in Figure 1a, four different electron-withdrawing/donating substituents (**1**: methoxy, **2**: tert-butyl, **4**: fluoro, and **5**: trifluoromethyl, respectively) were introduced into 3,6-diphenyl-1,2,4,5-tetrazine (**3, DPT**). The designed s-tetrazines were synthesized by a modified Pinner method using the corresponding 4-substituted benzonitriles as their respective starting materials [26,27], and their structures were fully characterized by ^1^H- and ^13^C-NMR and elemental analysis. The detailed synthetic procedures with the structure characterization data are described in the Experimental section.

To predict the change in the FMO energy levels of the s-tetrazines by the substitution, computational calculations were conducted by using the DFT method with 6–31+G(d) basis set and B3LYP functionals in the gas phase. The LUMO levels of the s-tetrazines were calculated to be −2.71, −2.80, −2.96, −3.17, and −3.50 eV for **1**, **2**, **3**, **4**, and **5**, respectively (Table 1). As expected, the methoxy (**1**) and t-butyl (**2**) groups destabilized the LUMO level, while F (**4**) and CF_3_ (**5**) groups stabilized it compared to that of DPT.

### 2.2. Electrochemical Properties

**Figure 2:**
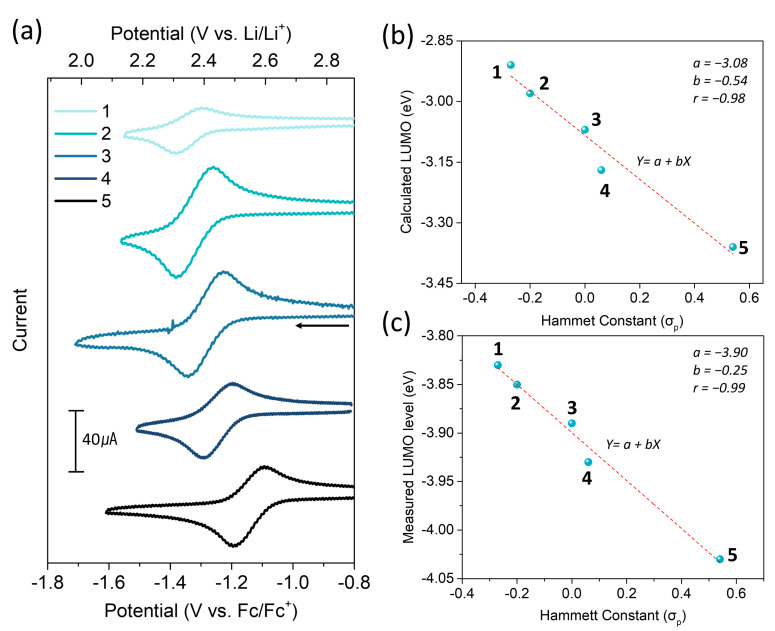
(**a**) Cyclic voltammetry (CV) of the s-tetrazines in 0.1 M [NBu_4_][PF_6_] acetonitrile (CH_3_CN) solution. Scan rate (v) was 50 mV s^−1^ with a 3 mm glassy carbon working electrode. The concentration of the s-tetrazine derivatives was 2 × 10^−3^ M in CH_3_CN except for **1** (1 × 10^−3^ M), which has lower solubility in CH_3_CN than the others. The linear fitting of (**b**) the calculated LUMO levels of the s-tetrazines in solvated system vs. the Hammett constant of substituents and (**c**) the measured LUMO levels by CV vs. Hammett constant.

The CV of the s-tetrazines was measured in acetonitrile (MeCN) solutions with 0.1 M tetrabutylammonium hexafluorophosphate (TBAHFP) as a supporting electrolyte. The cathodic CV scans showed quasi-reversible one-electron redox reactions for all s-tetrazines, and the reduction potentials (E_1/2_) were measured to be −1.34, −1.32, −1.28, −1.25 and −1.14 vs. Fc/Fc^+^ for **1**, **2**, **3**, **4**, and **5**, respectively, at a scan rate of 50 mV s^−1^ (Figure 2a). The anodic CV scans were also performed, but the oxidation of the s-tetrazines was not reversible (Figure S1). The LUMO and HOMO energy levels were obtained based on the formal potential of the ferrocene/ferrocenium couple [28] and displayed in Figure S2 and Table 1. It should be noted that the measured FMO levels correlated with the FMO trend calculated by DFT as well as the Hammett constant (Figure 2c and Figure S3) [29].

To examine the kinetics of the s-tetrazines, additional cathodic CV scans were performed at various scan rates. As shown in Figures S4 and S5, the s-tetrazines showed larger current values as the scan rate increased due to a decrease in the diffusion layer thickness. It was found that the current densities at anodic and cathodic peaks were proportional to square root of the scan rate (see Figure S6). Thus, the diffusion coefficients D (cm^2^ s^−1^) were calculated from Randles–Sevcik equation [30] as follows (Equation (1)).

(1) $$i_{p}=\left(2.69\;\times 10^{5}\right)n^{3/2}\cdot A\cdot D^{1/2}\cdot C\cdot v^{1/2}$$

where, $i_{p}\;$(*A*) is the peak current, *n* is the number of electrons transferred in the redox event, *A* (cm^2^) is the electrode surface area, C (mol cm^−3^) is the bulk concentration of the analyte, and v (V s^−1^) is the scan rate. The calculated *D* of the s-tetrazines were 1.63–3.63 × 10^−5^ cm^2^ s^−1^ (see Table 2 and Table 3).

Then, the heterogeneous electron transfer rate constant (*k*_0_) of the s-tetrazines were estimated using the following equations based on the Nicholson method [30,31].

(2) $$\varphi =\frac{{\left(D_{O}/D_{R}\right)}^{\alpha /2}k_{0}}{{\left(\pi D_{O}fv\right)}^{\frac{1}{2}}}$$

(3) f=F/RT

where φ is the Nicholson dimensionless number, which is a function of the peak-to-peak separation from a CV curve, *D_o_ and D_R_* is the diffusion coefficient of the oxidized and reduced species, respectively, α is the transfer coefficient, v is the scan rate, *F* is the Faraday constant (96,485 C mol^−1^), *R* is the ideal gas constant (8.314 J mol K^−1^), and *T* is the absolute temperature in K. Typically, the reduced species of the analyte were assumed to have the same diffusion coefficient with the oxidized one due to negligible molecular weight change during the redox reaction. Then, Equation (2) can be simplified as follows:

(4) $$\varphi =k_{0}{\left(\pi Dfv\right)}^{\left(-1/2\right)}\;$$

The *k*_0_ calculated from the plots of φ versus $v^{\left(-1/2\right)}$ (see Figures S4 and S6) was 2.55 × 10^−3^ cm s^−1^ for **3**, and the other s-tetrazines also showed high *k_0_* values (2–8 × 10^−3^ cm s^−1^, see Table 3). It is noteworthy that the s-tetrazines have several orders of magnitude faster *k_0_* values than V^2+^/V^3+^ (*k*_0_ = 10^−4^–10^−6^ cm s^−1^) redox couple [32], which is a commercialized anolyte for a redox flow battery (RFB), suggesting great promise of s-tetrazines not only as an active material for a metal-ion battery but also as an anolyte for a RFB.

### 2.3. Reduction Potential Prediction by DFT Calculation

To predict the change in the reduction potential depending on the substituents, we conducted computational calculations on the one-electron reduction reaction of the s-tetrazines in the solvated phase using the PCM model of DFT calculation method (The detailed calculation process is fully described in the Experimental section). We first investigated the free energy changes of the reduction reaction to form anions without a Li-ion insertion. The calculated Gibbs free energies of the s-tetrazines at the optimized geometries in the neutral and anion states are summarized in Tables S1–S3.

**Figure 3:**
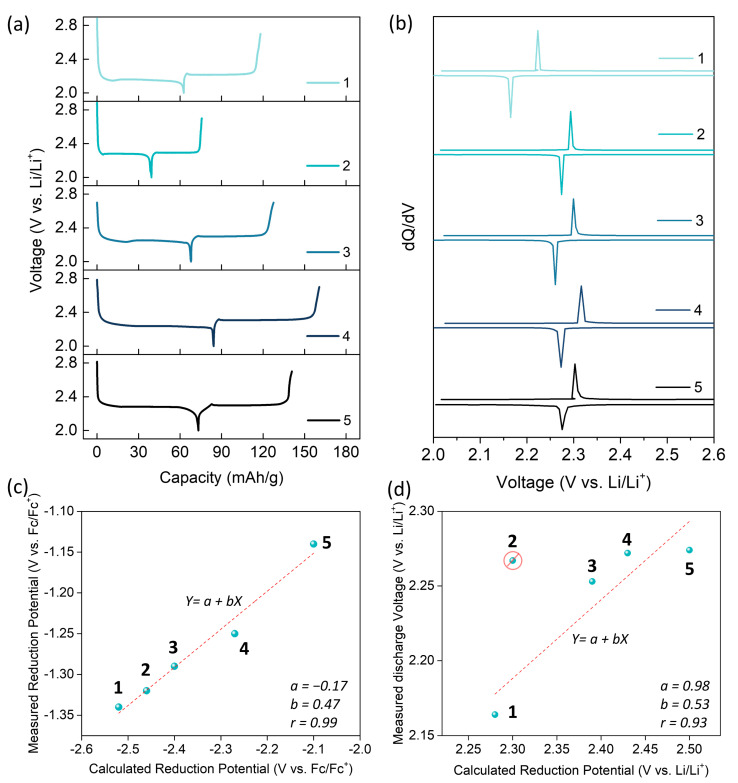
(**a**) The galvanostatic charge/discharge profiles for the s-tetrazine electrodes at 0.1 C rate. (**b**) The dQ/dV plots of the s-tetrazine electrodes. The correlation graphs about (**c**) the measured reduction potentials in CV vs. the calculated reduction potentials without considering Li-ion insertion by the DFT method and (**d**) the measured discharge voltages in Li-ion cells vs. the calculated reduction potentials considering Li-ion insertion. The dashed red lines in (**c**) and (**d**) indicate linear fitting curves and the regression coefficients are presented in the graphs. In graph (**d**), the point for the **2** electrode was excepted as an outlier for the linear regression because of the statistical reasons. See Figure S15 for the detailed analysis.

In the neutral states, it was found that the tetrazine core and the two peripheral benzene rings were coplanar regardless of the substituents (Figures S7–S11 in Supplementary Materials). Interestingly, upon reduction, the s-tetrazine anions showed no significant changes in the geometries. The LUMO orbitals of the neutral states and the spin densities of anion states were located on the tetrazine core regardless of the substituent (Figure S15). The reduction potentials were calculated to be −2.52, −2.46, −2.40, −2.27, and −2.10 V vs. Fc/Fc^+^ for **1**, **2**, **3**, **4,** and **5**, respectively. As expected, the calculated reduction potentials showed the same trend with the measured reduction potentials from the solution CV (see Figure 3c).

Next, we considered the insertion of a Li-ion to the reduced s-tetrazines for charge compensation. In the optimized geometries, the inserted Li cation was located at the one side of the tetrazine core in the same plane to form coordinate bonds with the two nitrogen atoms of the core ring (r _N-Li_ = 1.95–1.96 Å, Figures S7–S11). However, in contrast to the anion case above, it was found that slight distortion was generated between the tetrazine core and the two peripheral phenyl rings. The distorted angles were different depending on the substituents. The t-butyl-substituted s-tetrazine (**2**) exhibited the largest distorted angle (θ_1,2_ = 12.5°), while the CF_3_-substituted one (**5**) had virtually planar geometry (θ_1,2_ = 0.8°). When considering the Li-ion insertion, the reduction potentials of **1**, **2**, **3**, **4**, and **5** were calculated to be 2.28, 2.30, 2.40, 2.43, and 2.50 V vs. Li/Li^+^, respectively. However, it should be noted that the trend of reduction potential change depending on the substituents was unaffected by the Li-ion insertion.

### 2.4. CV and Galvanostatic Test in Coin Cells

To evaluate the electrochemical properties of the s-tetrazines in Li-ion cells, we fabricated composite electrodes composed of the s-tetrazines as an active material, Super-P as a conductive additive, and PVDF as a binder, respectively. In Li-ion cells, CV scans of the s-tetrazine electrodes were performed at various scan rates (Figures S12 and S13). Based on the Randles–Sevcik equation, the diffusion coefficient of Li-ion (*D_Li_*) in the s-tetrazine electrodes were calculated to be 7.30 × 10^−10^–2.50 × 10^−9^ cm^2^ s^−1^ for the cathodic peaks and 9.66 × 10^−10^–3.43 × 10^−9^ cm^2^ s^−1^ for the anodic peaks, respectively (see Figure S13 and Table 3), which are much higher values than those of conventional cathode materials for the Li-ion battery such as LCO (10^−11^–10^−13^ cm^2^ s^−1^) [33,34] and LFP (10^−13^–10^−14^ cm^2^ s^−1^) [35,36]. Typically, many organic electrode materials including the s-tetrazines were found to have high *D_Li_* (>10^−9^ cm^2^ s^−1^) [8,37] than the inorganic materials.

Then, galvanostatic charge/discharge tests were carried out for the s-tetrazine electrodes in Li-ion coin cells. As shown in Figure 3a, the s-tetrazine electrodes showed a clear charge/discharge plateau with specific discharge capacities of 63, 39, 68, 84, and 73 mAh g^−1^ for **1**–**5** in the first cycle at 0.1 C, respectively. The C-rate for each electrode was calculated by their respective theoretical specific capacities (C_theo_ = 91, 77, 144, 99, and 72 mAh g^−1^ for **1**–**5**, respectively). The different capacity utilization of the s-tetrazine electrodes is likely attributed to their solubility difference in the electrolyte. Unfortunately, all s-tetrazine electrodes showed a gradual capacity decrease during the cycle test due to the dissolution of active materials into the electrolyte. However, their coulombic efficiencies were maintained to 100% after the first cycle (Figure S14), indicating that their redox reactions were reversible in Li-ion cells.

In the differential analysis (dQ/dV) graphs (Figure 3b), each s-tetrazine electrode showed a sharp charge/discharge peak. As expected, the discharge voltage of the s-tetrazine electrodes was gradually shifted from 2.17 V (**1**) to 2.28 V (**5**) vs. Li/Li^+^ depending on the electron-donating/withdrawing capabilities of the substituents except **2**. In contrast to the fact that the lowered redox potential was predicted by the DFT calculation (vide supra), the **2** electrode bearing t-butyl substituents showed 0.01 V higher discharge voltage than that of the **3** electrode (2.26 V). It is also contrary to the solution CV results measured with the TBA-salt electrolyte (see Section 2.2). This exception is most likely attributed to the fact that the **2** molecules may have different crystal structure from the other s-tetrazine derivatives due to its bulky substituents [38]. In the bulk electrode, there have been a few reports that the interaction between active materials and Li-ions as well as the crystal structure changes during redox reactions could affect the discharge voltage of active materials [4,39,40], which will be further studied in the future.

Nevertheless, excepting the electrode of molecule **2**, which comprises the exceptionally largest substituent group among others, the measured discharge voltages of the other four s-tetrazine electrodes clearly showed a linear relationship (Pearson’s r = 0.93) with the calculated reduction potentials by DFT method (see Figure S15 for the detailed analysis). It means that the redox potential of s-tetrazine can be tuned by substitution of electron-donating or withdrawing groups and also can be predicted by DFT calculation.

## 3. Experimental Section

Chemicals were purchased from commercial suppliers and used without further purification unless otherwise stated. 3,6-diphenyl-1,2,4,5-tetrazine (DPT), 4-(tert-butyl)benzonitrile and 4-fluorobenzonitrile were purchased from TCI (Seoul, Korea). Hydrazine monohydrate was purchased from Sigma-Aldrich (Seoul, Korea). 4-methoxy benzonitrile, 4-(trifluoromethyl)benzonitrile and sulfur were purchased from Thermo Fisher Scientific (Incheon, Korea). Reactions were monitored using thin-layer chromatography (TLC) with commercial TLC plates (silica gel 60 F254) and silica gel column chromatography was performed with silica gel 60 (particle size 0.063–0.200 mm) from Sigma-Aldrich (Seoul, Korea). ^1^H-NMR and ^13^C-NMR spectra were recorded on a Bruker Avance III HD (300 Hz) and Avance III 500 (500 Hz), respectively. Elemental analyses were carried out using a Flash2000 elemental analyzer (Thermo Fisher Scientific).

### 3.1. Synthesis of 1

Hydrazine monohydrate (22 mL, 421 mmol) was added dropwise to a solution of 4-methoxy benzonitrile (8 g, 60 mmol) and sulfur (1.92 g, 60 mmol) in ethanol (15 mL). The reaction mixture was heated at reflux during overnight. After the reaction finished, the mixture was cooled to room temperature and the precipitate was quickly filtered and dried to afford crude dihydro tetrazines. The crude material (4.59 g, 15 mmol) was dissolved in dichloromethane (15 mL) and a solution of sodium nitrite (5.34 g, 77 mmol) in water (100 mL) was added to the mixture. The mixture was cooled to 0 °C and acetic acid (2.5 mL, 43 mmol) was added dropwise. The reaction mixture was stirred at room temperature during overnight. The organic layer was extracted and dried with MgSO_4_. The crude product was purified by column chromatography (dichloromethane:n-Hex = 1:2, silica gel), yielding a dark red solid. Yield: 2.3 g (26%). ^1^H NMR (300 MHz, CDCl3, δ): 8.58 (d, J = 8.70 Hz, 4H), 7.10 (d, J = 8.73 Hz, 4H), 3.93 (s, 6H); ^13^C NMR (125 MHz, CDCl3, δ): 163.43, 163.40, 129.77, 124.60, 114.94, 55.73. Anal. Calcd for C_16_H_14_N_4_O_2_: C, 65.30; H, 4.79; N, 19.04; O, 10.87. Found: C, 65.28; H, 4.77; N, 19.04; O, 10.93.

### 3.2. Synthesis of 2

Hydrazine monohydrate (11 mL, 209 mmol), was added dropwise to a solution of 4-(tert-butyl)benzonitrile (4.75 g, 30 mmol) and sulfur (0.96 g, 30 mmol) in ethanol (15 mL). The reaction mixture was heated at reflux during overnight. After the reaction finished, the mixture was cooled to room temperature and the precipitate was quickly filtered and dried to afford crude dihydro tetrazines. The crude material (3.89 g, 11 mmol) was dissolved in dichloromethane (15 mL) and a solution of sodium nitrite (3.84 g, 56 mmol) in water (150 mL) was added to the mixture. The mixture was cooled to 0 °C and acetic acid (1.79 mL, 31 mmol) was added dropwise. The reaction mixture was stirred at room temperature during overnight. The organic layer was extracted and dried with MgSO_4_. The crude product was purified by column chromatography (dichloromethane:n-Hex = 1:19 to 1:4, silica gel), yielding a pink solid. Yield: 0.47 g (10%). ^1^H NMR (300 MHz, CDCl3, δ): 8.58 (d, J = 8.61 Hz, 4H), 7.64 (d, J = 8.59 Hz, 4H), 1.41 (s, 18H); ^13^C NMR (125 MHz, CDCl3, δ): 163.95, 156.51, 129.25, 127.92, 126.54, 35.36, 31.36. Anal. Calcd for C_22_H_26_N_4_: C, 76.27; H, 7.56; N, 16.17. Found: C, 76.31; H, 7.54; N, 16.18.

### 3.3. Synthesis of 4

Hydrazine monohydrate (8.93 mL, 173 mmol), was added dropwise to a solution of 4-fluorobenzonitrile (3 g, 25 mmol) and sulfur (0.8 g, 25 mmol) in ethanol (10 mL). The reaction mixture was heated at reflux during overnight. After the reaction finished, the mixture was cooled to room temperature and the precipitate was quickly filtered and dried to afford crude dihydro tetrazines. The crude material (3.29 g, 12 mmol) was dissolved in dichloromethane (20 mL) and a solution of sodium nitrite (4.17 g, 60 mmol) in water (200 mL) was added to the mixture. The mixture was cooled to 0 °C and acetic acid (1.94 mL, 34 mmol) was added dropwise. The reaction mixture was stirred at room temperature during overnight. The organic layer was extracted and dried with MgSO_4_. The crude product was purified by column chromatography (dichloromethane:n-Hex = 1:3, silica gel), yielding a purple solid. Yield: 1.2 g (36%). ^1^H NMR (300 MHz, CDCl3, δ): 8.65–8.69 (m, 4H), 7.28–7.34 (m, 4H); ^13^C NMR (125 MHz, CDCl3, δ): 167.07, 165.05, 163.34, 130.50, 128.14, 116.84. Anal. Calcd for C_14_H_8_F_2_N_4_: C, 62.22; H, 2.98; F, 14.06; N, 20.73. Found: C, 62.29; H, 3.00; N, 20.74.

### 3.4. Synthesis of 5

Hydrazine monohydrate (8.43 mL, 164 mmol), was added dropwise to a solution of 4-(trifluoromethyl)benzonitrile (4 g, 23 mmol) and sulfur (0.74 g, 23 mmol) in ethanol (10 mL). The reaction mixture was heated at reflux during overnight. After the reaction finished, the mixture was cooled to room temperature and the precipitate was quickly filtered and dried to afford crude dihydro tetrazines. The crude material (3.55 g, 10 mmol) was dissolved in dichloromethane (15 mL) and a solution of sodium nitrite (3.29 g, 48 mmol) in water (150 mL) was added to the mixture. The mixture was cooled to 0 °C and acetic acid (1.53 mL, 27 mmol) was added dropwise. The reaction mixture was stirred at room temperature during overnight. The organic layer was extracted and dried with MgSO_4_. The crude product was purified by column chromatography (dichloromethane:n-Hex = 1:5, silica gel), yielding a purple solid. Yield: 1.1 g (24%). ^1^H NMR (300 MHz, CDCl3, δ): 8.82 (d, J = 8.39 Hz, 2H), 7.91 (d, J = 8.41 Hz, 2H); ^13^C NMR (125 MHz, CDCl3, δ): 163.69, 134.98, 134.85, 134.60, 128.70, 126.59. Anal. Calcd for C_16_H_8_F_6_N_4_: C, 51.90; H, 2.18; F, 30.79; N, 15.13. Found: C, 52.04; H, 2.32; N, 15.14.

### 3.5. Electrochemical Measurement

Cyclic voltammetry (CV) was performed on a Princeton Applied Research Model 273a using a three-electrode beaker cell with an Ag wire in 0.01 M AgNO_3_ solution as a reference electrode, a glassy carbon disc (diameter = 3 mm) as a working electrode, and a platinum wire as a counter electrode, respectively. The redox potential of the reference electrode was calibrated using ferrocene/ferrocenium (Fc/Fc^+^) as an internal standard. 0.1 M tetrabutylammonium hexafluorophosphate (TBAHFP) was used as a supporting electrolyte. The concentration of the s-tetrazines solutions for the CV measurements was 2 × 10^−3^ M in acetonitrile (MeCN) except **1**. An MeCN solution containing 1 × 10^−3^ M of **1** used for the CV due to its low solubility.

### 3.6. Cathode Fabrication and Galvanostatic Test

Slurries of the active materials (**1**, **2**, **3**, **4,** and **5**), carbon black (Timcal Super P), and polyvinylidene fluoride (PVDF, Sigma Aldrich) in dimethylformamide (DMF, 99.5%, JUNSEI) were prepared with a weight ratio of 4:4:2. The slurries were stirred overnight at room temperature and then spread on aluminum foils by doctor blading. The electrodes were dried at 25 °C for 8 h in a vacuum oven and punched into circular discs to a diameter of 14 mm. Coin type CR2032 (Hohsen) cells were assembled with the fabricated cathodes, a Li-metal anode, and a polypropylene separator (Celgard 2400) in an Ar-filled glove box (Korea Kiyon KK-011-AS) in which moisture and oxygen levels were tightly regulated under 0.5 ppm. 2 M lithium bis(trifluoromethanesulfonyl) imide (LiTFSI) in a 1:1 (v/v) mixture of 1,3-dioxolane (DOL) and dimethoxy ethane (DME) with 1% LiNO_3_ is used for the electrolyte. The galvanostatic discharge/charge tests of the coin cells were performed on a battery cycler (Wonatech WBCS3000L) at 30 °C.

### 3.7. Theoretical Calculation

All density functional theory (DFT) calculations were carried out using Gaussian 09 quantum chemical package [41]. The geometry optimizations were performed using Becke-Lee-Yang-Parr (B3LYP) functionals and the 6–31G+(d) basis set. Vibrational frequency calculations were performed for the obtained structures at the same level to confirm the stable minima. The frontier molecular orbital (FMO) levels of the s-tetrazine molecules were calculated in the gas phase.

To calculate the reduction potential of the s-tetrazines, geometries of the neutral and the reduced molecules were optimized in the solvated phase using PCM model with the dielectric constant (ε) of 7.155 for DOL/DME (ε_DOL_ = 7.13 and ε_DME_ = 7.18) [4,42,43,44,45,46]. Note that, to reduce the calculation cost, an average ε value was employed for the 1:1 DOL/DME mixture solvent without solvation cavity modification. Then, the reduction potential was calculated using the following formula:

*E*_red_ = − (*G*_anion_ − *G*_neutral_)/*nF*, (5)

where *G*_neutral_ and *G*_anion_ are the Gibbs free energies of the s-tetrazines at the neutral and anion state, respectively; *n* is the number of electrons involved in the reaction; *F* is the Faraday constant.

To consider the effect of cation insertion, a Li cation was added to the optimized anion form of the s-tetrazines. The reduction potentials were calculated using the following formula:

*E*_red_ = − (*G*_lithiated form_ − *G*_neutral_ − *G*_Li cation_)/*nF*, (6)

where *G*_lithiated form_ and *G*_Li cation_ are Gibbs free energies of the lithiated s-tetrazine molecules and a Li cation, respectively. The calculated reduction potentials were shifted by 1.917 V with respect to Li/Li^+^ as calculated.

## 4. Conclusions

In summary, we investigated the substituent effect on the redox potentials and discharge voltages of the s-tetrazine derivatives. The theoretical DFT calculation and the practical CV measurement clearly revealed that the electron-donating substituents (i.e., MeO and t-Bu) destabilized the FMO energy levels to lower the redox potential of the s-tetrazine, while the electron-withdrawing groups (i.e., F and CF_3_) stabilized their FMO energy levels to elevate their redox potential. It should also be noted that the potential changes depended on the electron-donating/withdrawing capabilities of the substituents, which was shown by a correlation with the Hammett constant. Most importantly, the discharge voltages of the s-tetrazine electrodes were also varied by the substituents, and the trend of voltage change well correlated with the calculated values from the DFT except one outlier. Although the tuned voltage of the DPT derivatives in this study was rather small, it is worth noting that introducing substituents clearly affected the discharge voltages and the change could be well predicted by theoretical calculation.

On the other hand, it was revealed that the s-terazines possess high heterogenous electron transfer rate constants (*k*_0_) by the solution CV measurements. In addition, in the Li-ion coin cells, it was observed that the Li-ion diffusion in the s-tetrazine electrodes was much faster than conventional LCO and LFP electrodes. These results clearly indicate that the s-tetrazine redox center is a promising candidate for an active material with high power capability in RFBs as well as metal-ion batteries.

## Figures and Tables

**Table 1:** The HOMO, LUMO levels and the redox potentials of s-tetrazines.

	^a^ HOMO (ev)	^a^ LUMO (ev)	^b^ Reduction Potential (E_1/2_) (V vs. Fc/Fc^+^)	^c^ Oxidation Potential (E_onset_) (V vs. Fc/Fc^+^)	^d^ HOMO (ev)	^d^ LUMO (ev)
**1**	−6.02	−2.71	−1.34	1.29	−6.39	−3.76
**2**	−6.31	−2.80	−1.32	1.42	−6.52	−3.78
**3**	−6.46	−2.96	−1.28	1.48	−6.58	−3.82
**4**	−6.67	−3.17	−1.25	1.51	−6.61	−3.85
**5**	−7.01	−3.50	−1.14	1.60	−6.70	−3.96

**^a^** The calculated FMO levels by DFT method. **^b^** The measured half-wave potentials of the reduction reaction by CV. **^c^** The measured onset potentials of the oxidation reaction by CV. **^d^** The measured HOMO and LUMO levels calibrated by HOMO level of ferrocene (5.1 eV).

**Table 2:** Diffusion coefficients (*D*) and heterogeneous electron transfer rate constants (*k*_0_) of tetrazines.

	*D* (cm^2^ s^−1^)	*k_0_* (cm s^−1^)
**1**	3.63 × 10^−5^	8.50 × 10^−3^
**2**	1.63 × 10^−5^	2.76 × 10^−3^
**3**	2.19 × 10^−5^	2.55 × 10^−3^
**4**	2.06 × 10^−5^	5.58 × 10^−3^
**5**	2.02 × 10^−5^	5.06 × 10^−3^

**Table 3:** Li-ion diffusion coefficients (*D_Li_*) and the discharge voltages of the s-tetrazine electrodes.

	*D_Li_* (cm^2^ s^−1^) for Cathodic Peak	*D_Li_* (cm^2^ s^−1^) for Anodic Peak	^a^ Discharge Voltage (V vs. Li/Li^+^)
**1**	1.51 × 10^−9^	2.02 × 10^−9^	2.17
**2**	7.30 × 10^−10^	9.66 × 10^−10^	2.27
**3**	1.34 × 10^−9^	1.60 × 10^−9^	2.26
**4**	2.50 × 10^−9^	3.43 × 10^−9^	2.27
**5**	1.45 × 10^−9^	3.08 × 10^−9^	2.28

**^a^** The discharge voltages obtained from the dQ/dV plots.

## Data Availability

The data presented in this study are available in article.

## Supplementary Materials

The following are available online. Figure S1: The anodic CV of the s-tetrazines measured in acetonitrile solutions with 0.1 M tetrabutylammonium hexafluorophosphate (TBAHFP) as a supporting electrolyte and an Ag wire in 0.01 M AgNO3 solution as a reference electrode at a scan rate of 50 mV s^−1^, Figure S2. The HOMO and LUMO energy levels of the s-tetrazines obtained from solution CV, Figure S3. The linear fitting of the measured (a) LUMO level and (b) HOMO level by CV vs. the calculated FMO levels of the s-tetrazines by DFT. (c) The linear fitting of the measured HOMO levels by CV vs. Hammett Constant, Figure S4. (a) The scan-rate-dependent CV of **3** in 0.1 M [NBu4][PF6] acetonitrile solution. (b) The peak current density of **3** vs. the square root of the scan rate from CV. (c) The graph of φ vs. v^−1/2^ by Nicholson method to calculate standard rate constant (k_0_), Figure S5. The scan-rate-dependent CV of (a) **1**, (b) **2**, (c) **4** and (d) **5** in 0.1 M [NBu_4_][PF_6_] acetonitrile solution, Figure S6. The peak current density vs. the square root of the scan rate of (a) **1**, (c) **2**, (e) **4** and (g) **5**. The graph of φ vs. v^−1/2^ by Nicholson method to calculate standard rate constant of (b) **1**, (d) **2**, (f) **4** and (h) **5,** Figure S7. The optimized geometries and total energies in Hartrees of (a) the neutral, (b) the reduced, and (c) the lithiated **1** in the solvated phase. The top and side views are shown at left and right panels, respectively, Figure S8. The optimized geometries and total energies in Hartrees of (a) the neutral, (b) the reduced, and (c) the lithiated **2** in the solvated phase. The top and side views are shown at left and right panels, respectively, Figure S9. The optimized geometries and total energies in Hartrees of (a) the neutral, (b) the reduced, and (c) the lithiated **3** in the solvated phase. The top and side views are shown at left and right panels, respectively, Figure S10. The optimized geometries and total energies in Hartrees of (a) the neutral, (b) the reduced, and (c) the lithiated **4** in the solvated phase. The top and side views are shown at left and right panels, respectively, Figure S11. The optimized geometries and total energies in Hartrees of (a) the neutral, (b) the reduced, and (c) the lithiated **5** in the solvated phase. The top and side views are shown at left and right panels, respectively, Figure S12. (a) Cyclic Voltammetry of the **3** electrode with different scan rates and (b) the plot of peak current density vs. the square root of the scan rate for obtaining Li-ion diffusion coefficient, Figure S13. Cyclic Voltammetry of the tetrazine electrodes with different scan rates and the plot of cathodic and anodic peak current density vs. the square root of the scan rate. (a) and (b) for **1** electrode, (c) and (d) for **2** electrode, (e) and (f) for **4** electrode, (g) and (h) for **5** electrode, respectively, Figure S14. The cycle retention and the corresponding coulomb efficiency of the s-tetrazine electrodes at 0.1 C, Figure S15. The correlation graphs about the measured discharge voltages in Li-ion cells vs. the calculated reduction potentials considering Li-ion insertion. The dashed red lines indicate linear fitting curves, and the corresponding regression coefficients are presented in the graphs. In the linear regression, all five points were used for (a), but the point 1 and the point 2 were excluded as an outlier for (b) and (d), respectively. The graph (c) is a magnified plot of the circled region in (b), Figure S16. The LUMO orbital of neutral s-tetrazines (left) and the spin density distribution of radical s-tetrazines (right) ((a) **1**, (b) **2**, (c) **3**, (d) **4** and (e) **5**), Figure S17. ^1^H and ^13^C NMR spectra of **1,** Figure S18. ^1^H and ^13^C NMR spectra of **2,** Figure S19. ^1^H and ^13^C NMR spectra of **4,** Figure S20. ^1^H and ^13^C NMR spectra of **5,** Table S1. Gibbs Free energies of a Li atom and Li cation, Table S2. Redox potentials of tetrazines without a Li-ion insertion, Table S3. Redox potentials of tetrazines with Li cation insertion.

## Sample Availability

Samples of the s-tetrazines are available from the authors.
