# Supplementary material for: Redox Potential Tuning of s-Tetrazine by Substitution of Electron-Withdrawing/Donating Groups for Organic Electrode Materials
Source: Molecules. 2021 Feb 8;26(4):894. doi: 10.3390/molecules26040894 (PMC7914744; doi:10.3390/molecules26040894)
Supplement: Supplementary file 1 [file molecules-26-00894-s001.pdf]

molecules

MDPI

Supporting Information for

Redox Potential Tuning of s-Tetrazine by Substitution of Electron-Wthdrawing/donating Groups for Organic Electrode Materials

Dong Joo Min [1], Kyunam Lee [1], Hyunji Park [1], Ji Eon Kwon [2,*] and Soo Young Park [1,*]

[1] Lab for Supramolecular Optoelectronic Materials (LSOM), Research Institute of Advanced Materials (RIAM), Department of Materials Science and Engineering, Seoul National University, 1 Gwanak-ro, Gwanak-gu, Seoul 08826, Republic of Korea

[2] Functional Composite Materials Research Center, Institute of Advanced Composite Materials, Korea Institute of Science and Technology (KIST), 92 Chudong-ro, Bongdong-eup, Wanju-gun, Jeonbuk 55324, Republic of Korea

* Correspondence: jekwon@kist.re.kr (J.E.K.), parksy@snu.ac.kr (S.Y.P.)

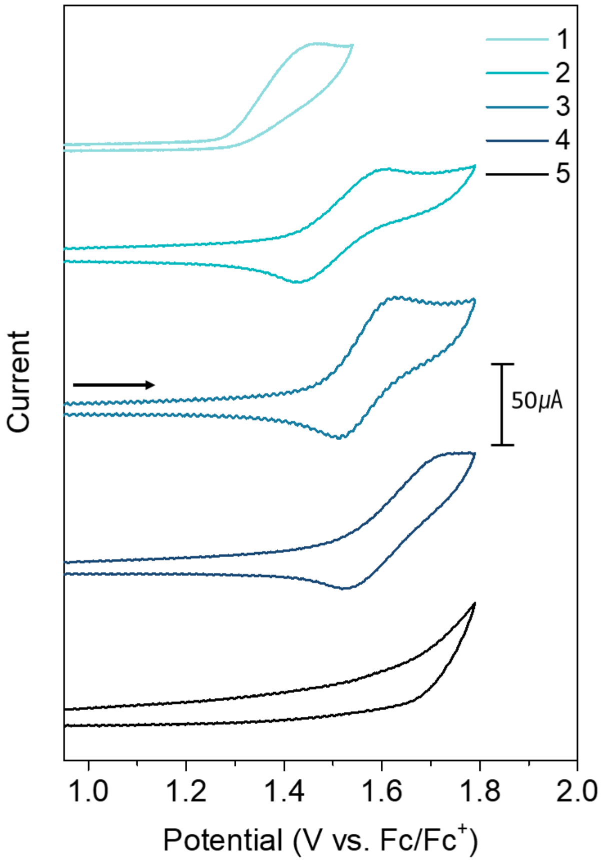

Figure S1. The anodic CV of the s-tetrazines measured in acetonitrile solutions with 0.1 M tetrabutylammonium hexafluorophosphate (TBAHFP) as a supporting electrolyte and an Ag wire in 0.01 M AgNO3 solution as a reference electrode at a scan rate of 50 mV s^{-1}.

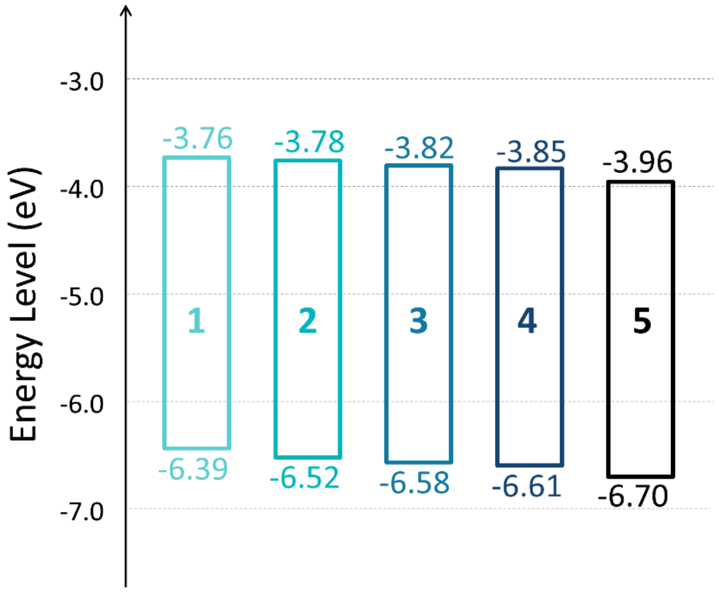

Figure S2. The HOMO and LUMO energy levels of the s-tetrazines obtained from solution CV.

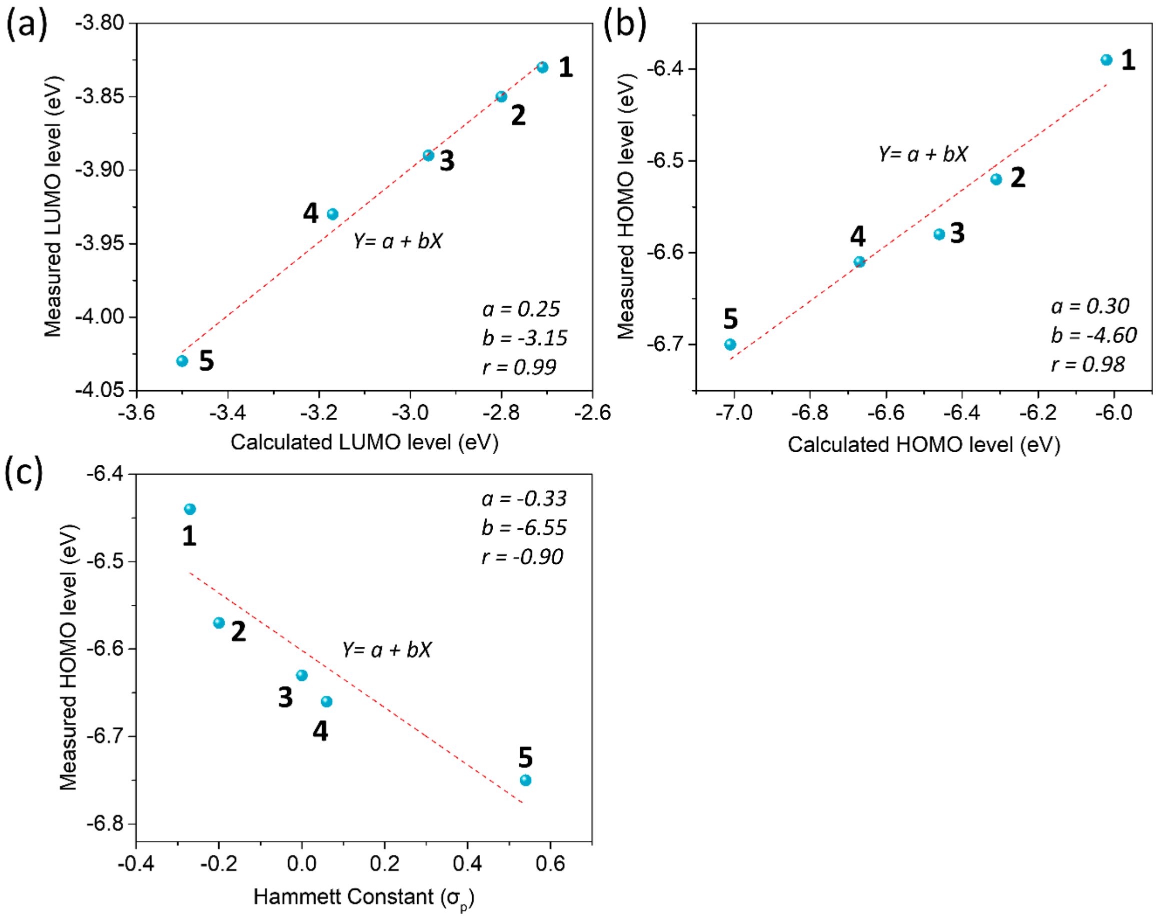

Figure S3. The linear fitting of the measured (a) LUMO level and (b) HOMO level by CV vs. the calculated FMO levels of the s-tetrazines by DFT. (c) The linear fitting of the measured HOMO levels by CV vs. Hammett Constant.

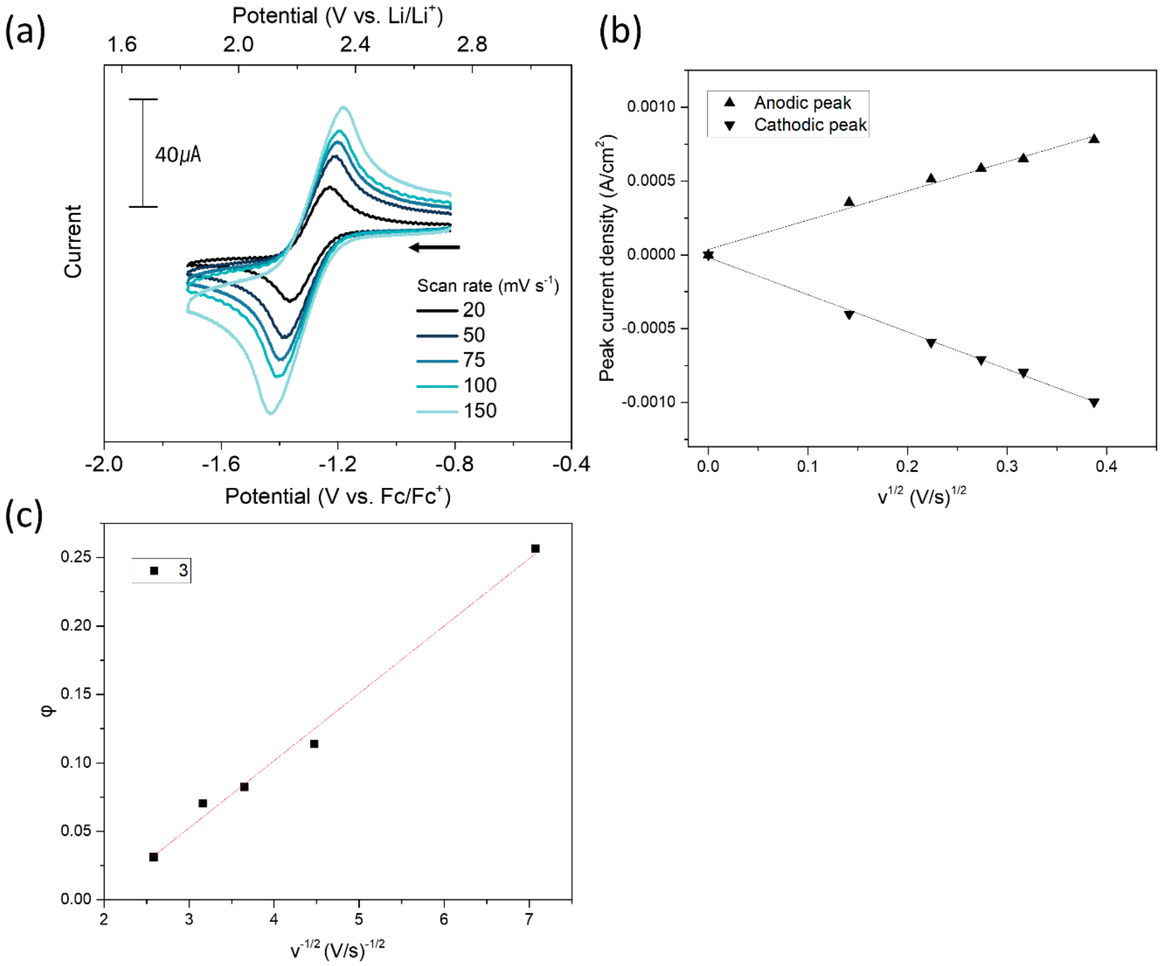

Figure S4. (a) The scan-rate-dependent CV of **3** in 0.1 M [NBu4][PF6] acetonitrile solution. (b) The peak current density of **3** vs. the square root of the scan rate from CV. (c) The graph of φ vs. $v^{-1/2}$ by Nicholson method to calculate standard rate constant (k_0).

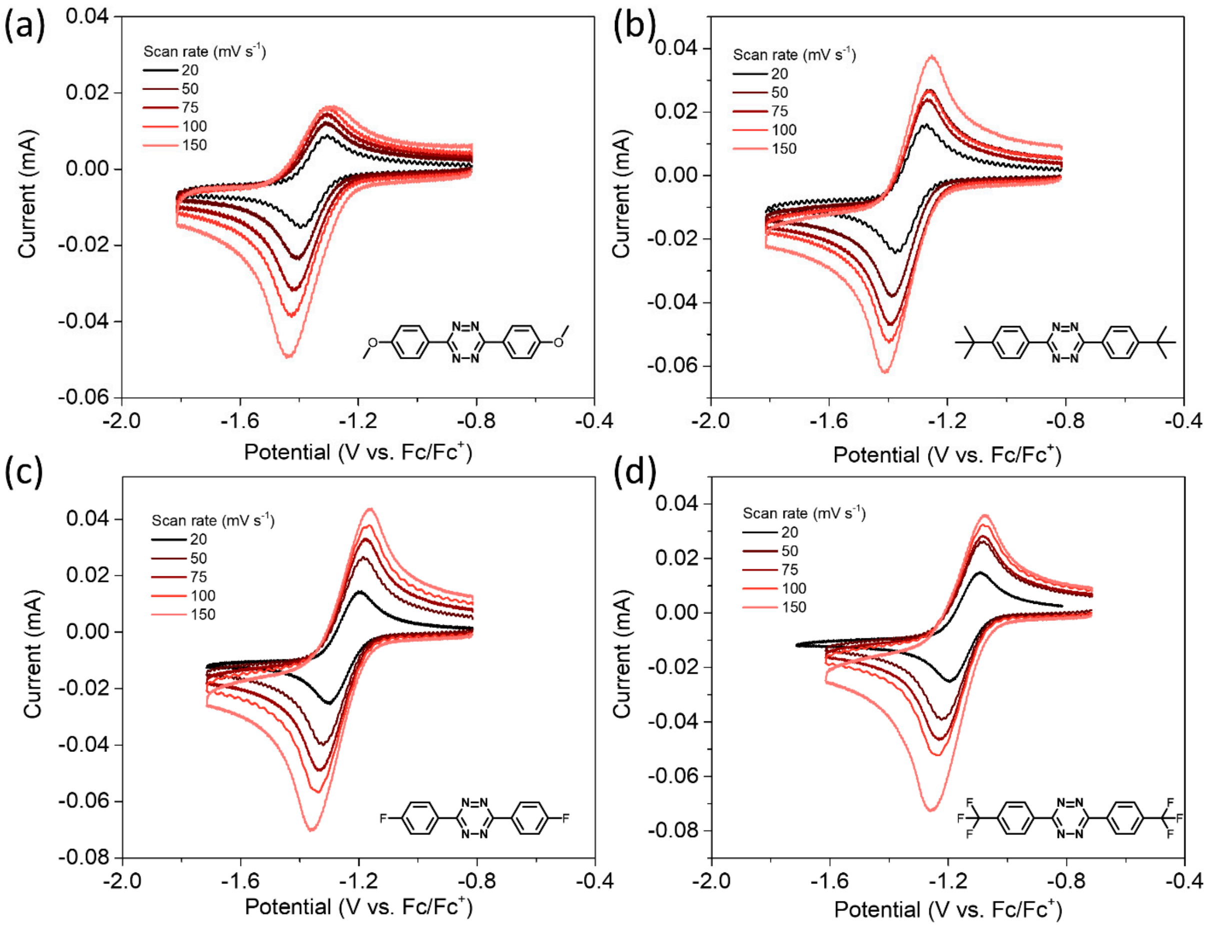

Figure S5. The scan-rate-dependent CV of (a) **1**, (b) **2**, (c) **4** and (d) **5** in 0.1 M $[NBu_4][PF_6]$ acetonitrile solution.

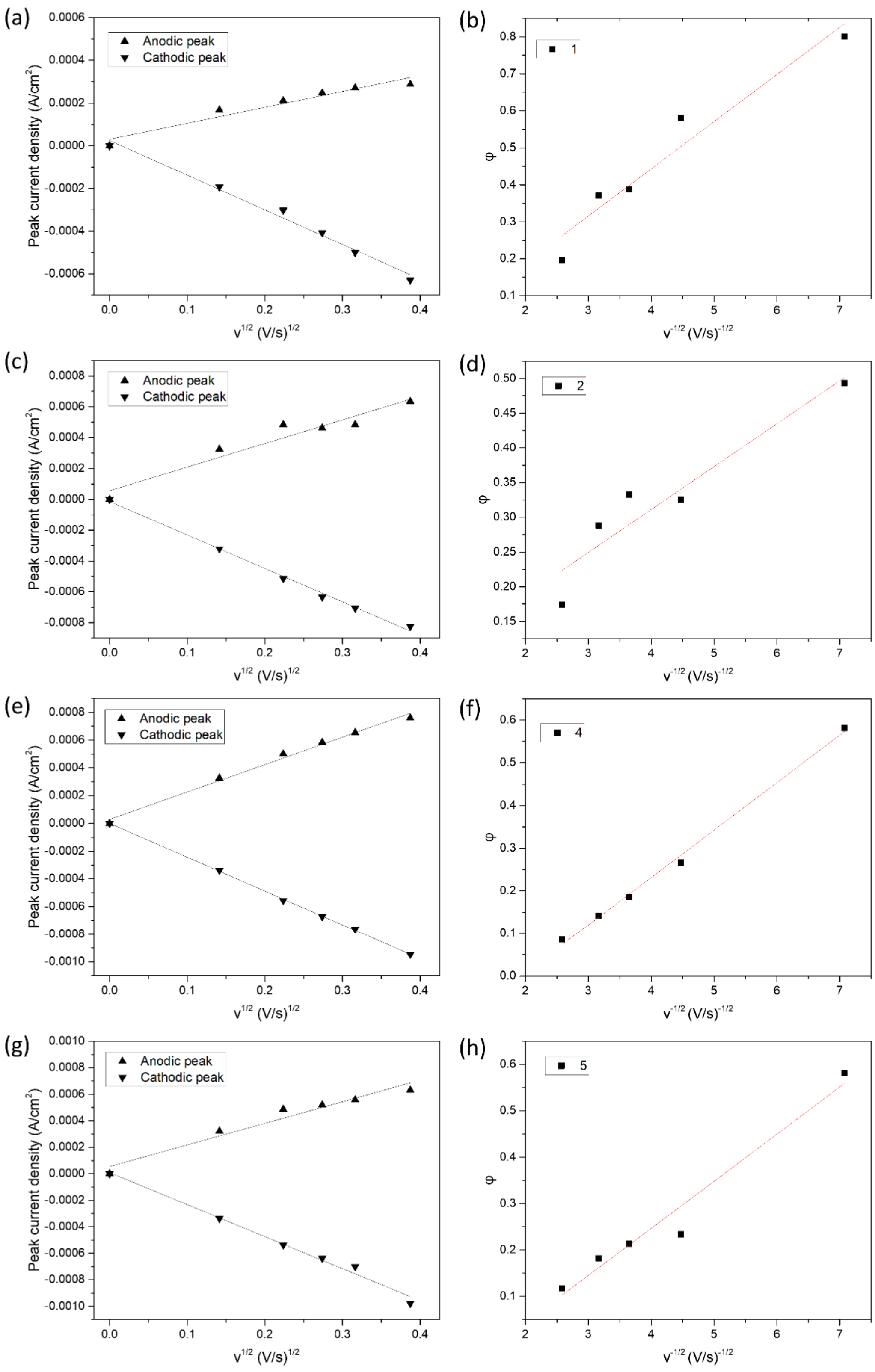

Figure S6. The peak current density vs. the square root of the scan rate of (a) **1**, (c) **2**, (e) **4** and (g) **5**. The graph of φ vs. $v^{-1/2}$ by Nicholson method to calculate standard rate constant of (b) **1**, (d) **2**, (f) **4** and (h) **5**.

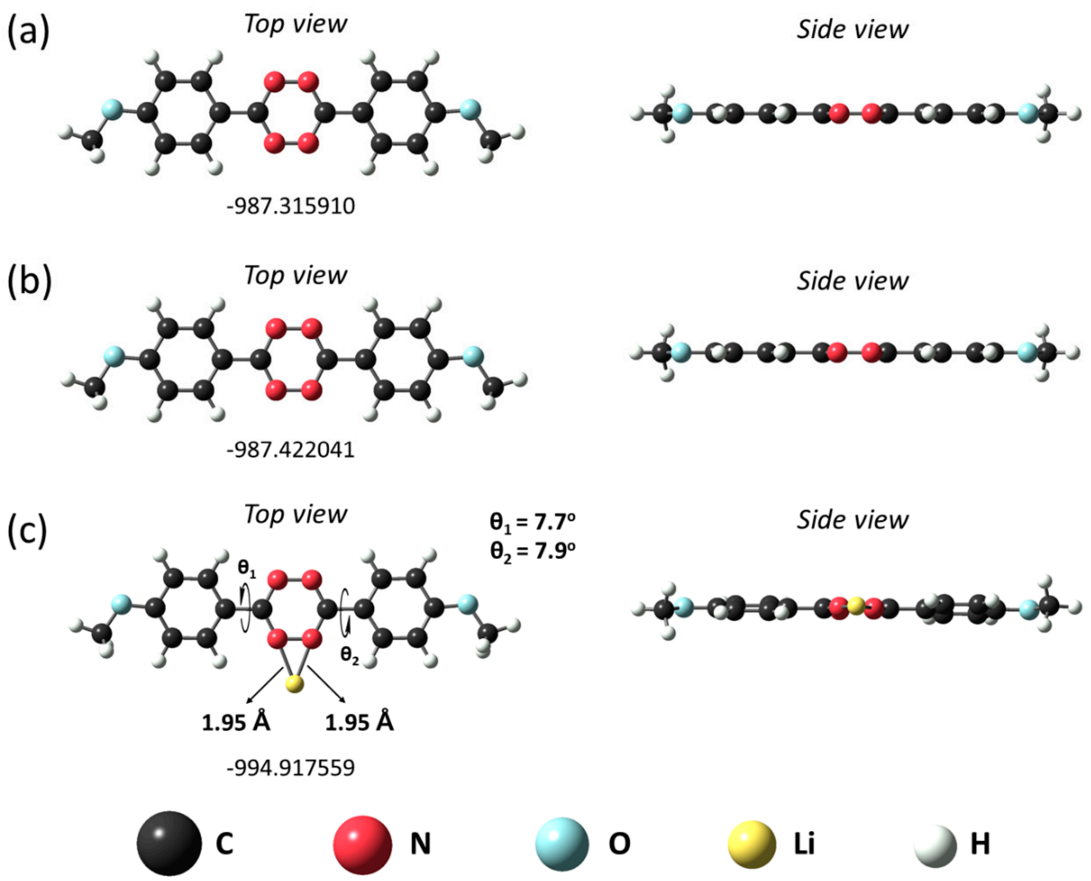

Figure S7. The optimized geometries and total energies in Hartrees of (a) the neutral, (b) the reduced, and (c) the lithiated **1** in the solvated phase. The top and side views are shown at left and right panels, respectively.

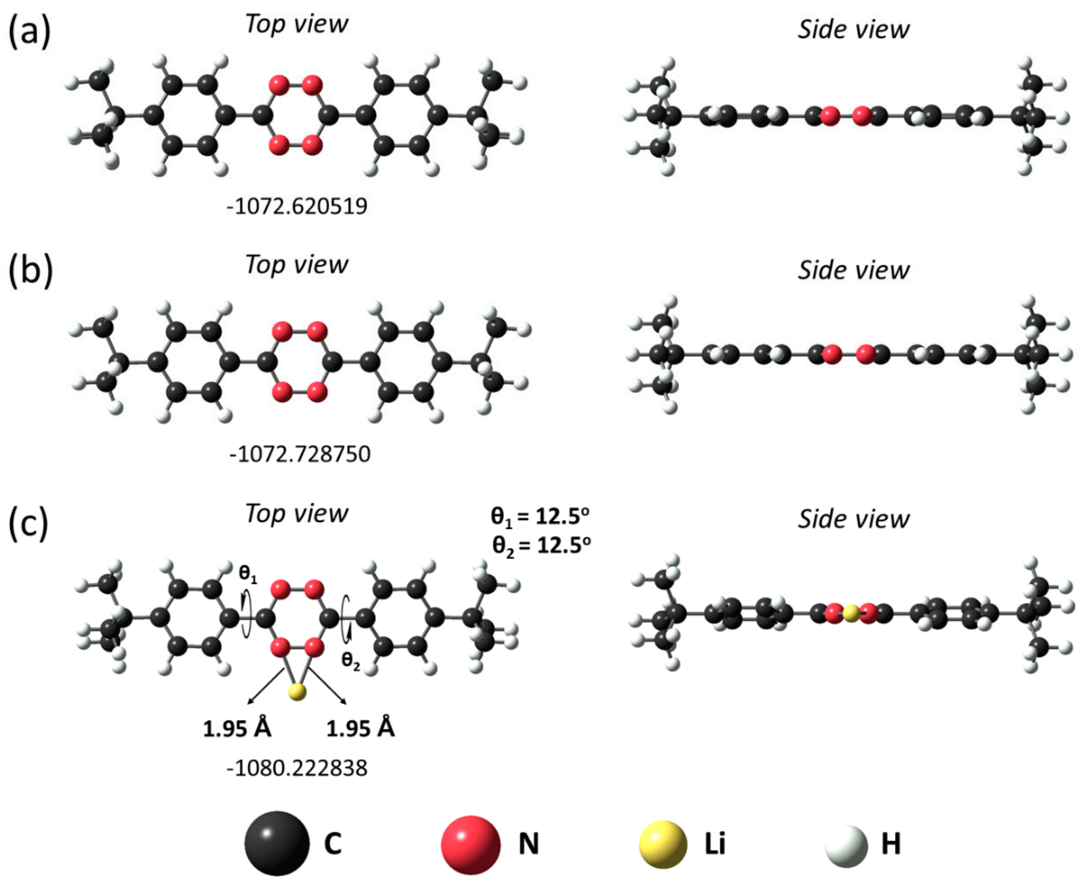

Figure S8. The optimized geometries and total energies in Hartrees of (a) the neutral, (b) the reduced, and (c) the lithiated **2** in the solvated phase. The top and side views are shown at left and right panels, respectively.

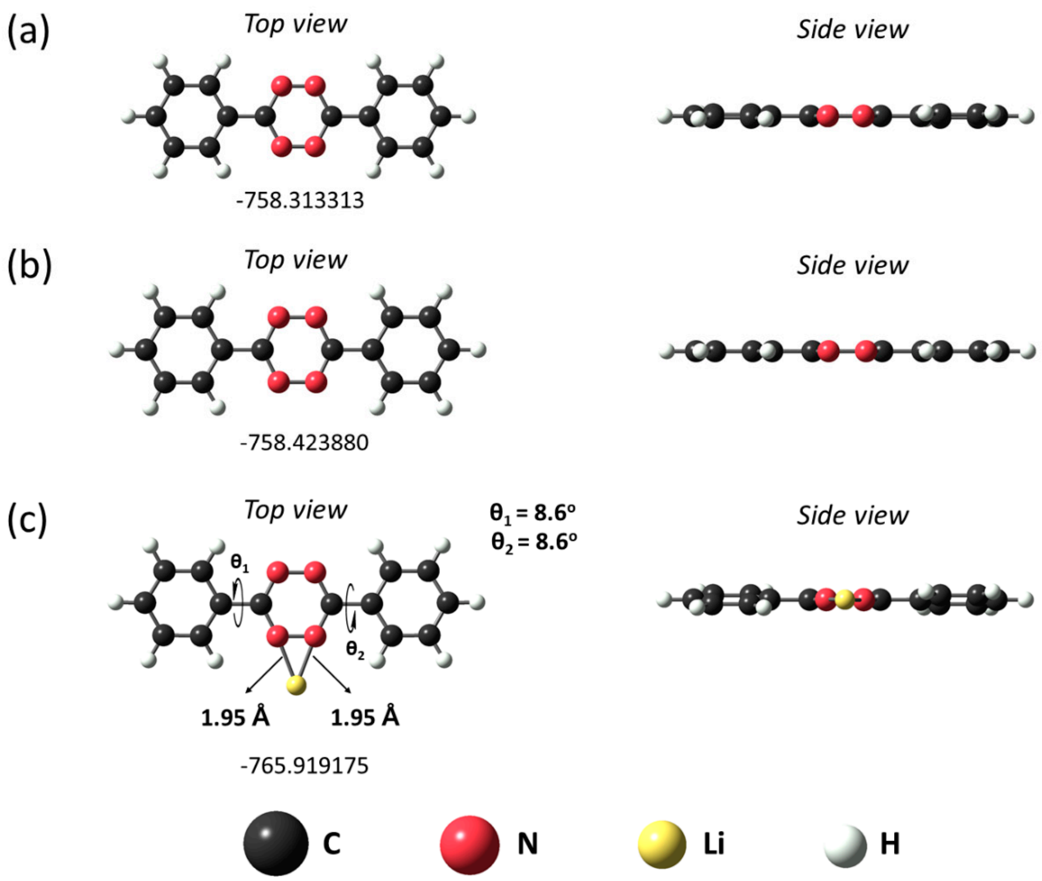

Figure S9. The optimized geometries and total energies in Hartrees of (a) the neutral, (b) the reduced, and (c) the lithiated **3** in the solvated phase. The top and side views are shown at left and right panels, respectively.

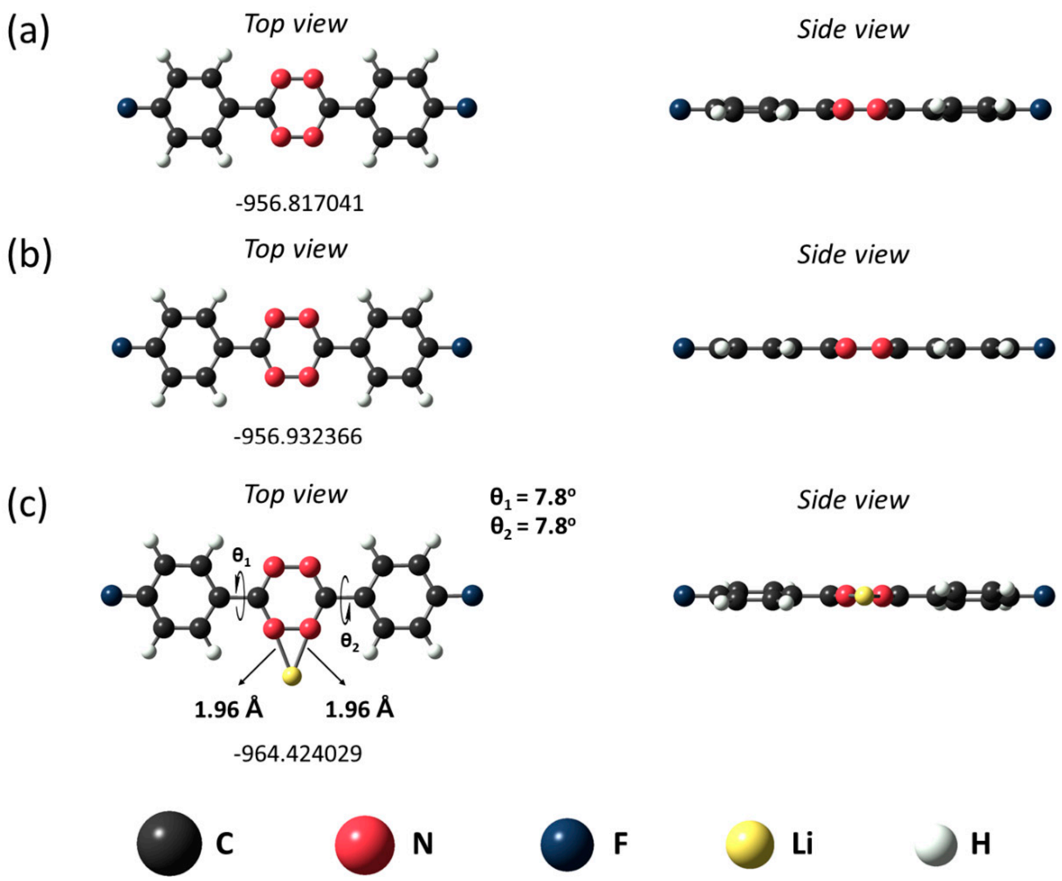

Figure S10. The optimized geometries and total energies in Hartrees of (a) the neutral, (b) the reduced, and (c) the lithiated **4** in the solvated phase. The top and side views are shown at left and right panels, respectively.

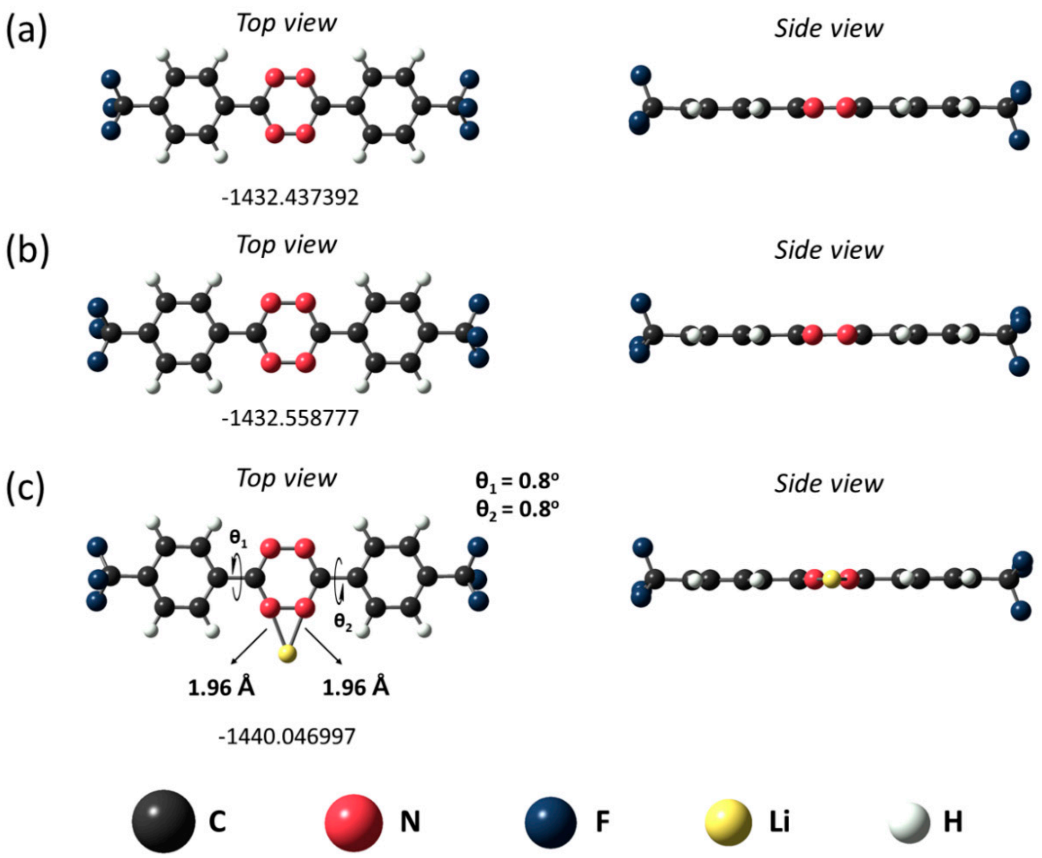

Figure S11. The optimized geometries and total energies in Hartrees of (a) the neutral, (b) the reduced, and (c) the lithiated **5** in the solvated phase. The top and side views are shown at left and right panels, respectively.

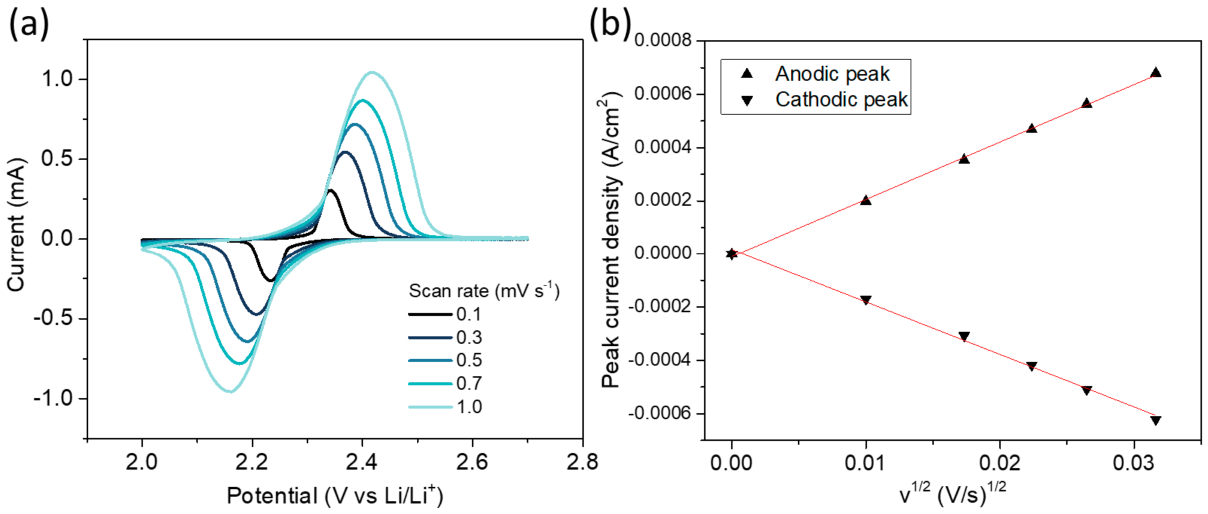

Figure S12. (a) Cyclic Voltammetry of the **3** electrode with different scan rates and (b) the plot of peak current density vs. the square root of the scan rate for obtaining Li-ion diffusion coefficient.

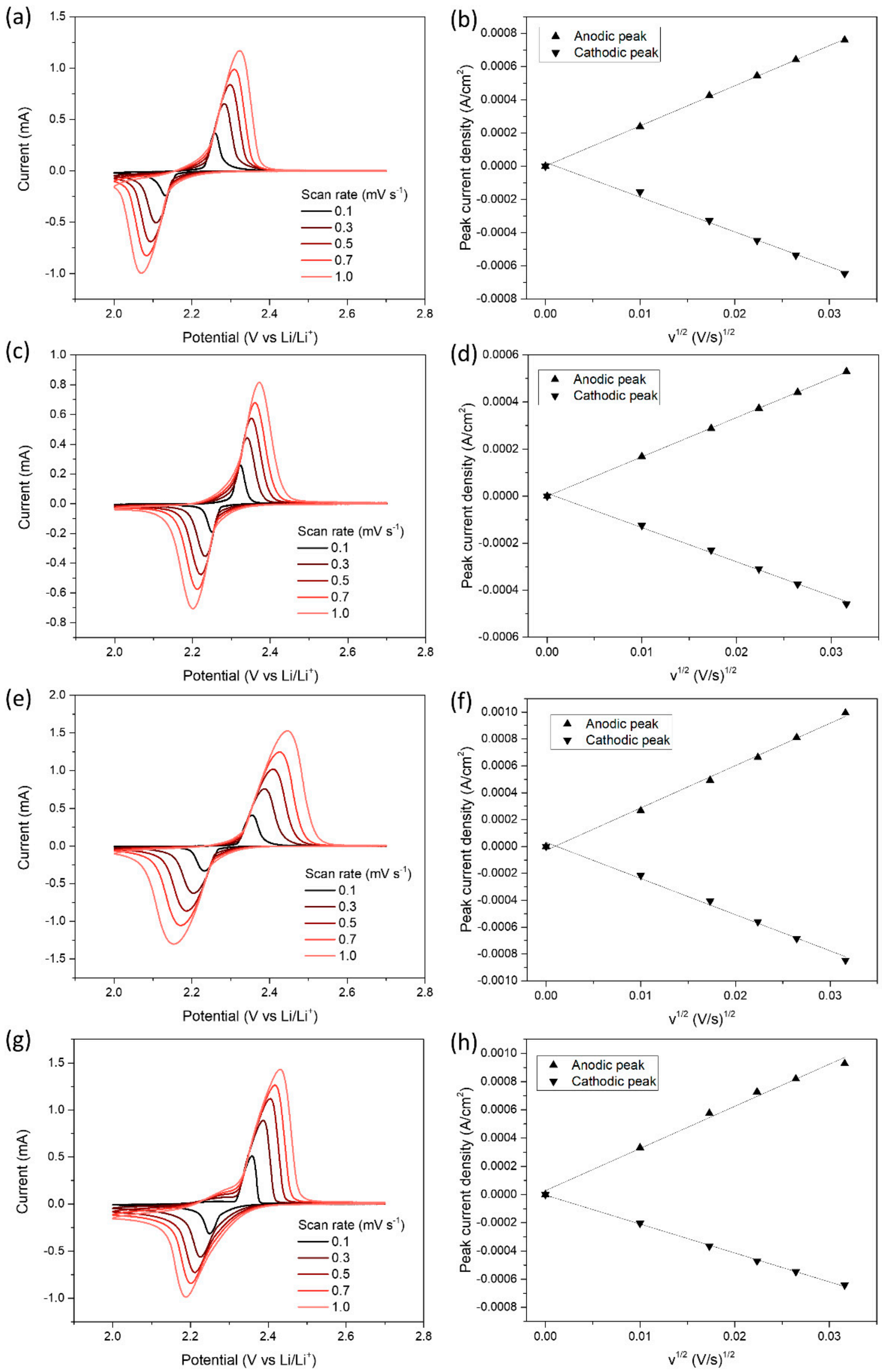

Figure S13. Cyclic Voltammetry of the tetrazine electrodes with different scan rates and the plot of cathodic and anodic peak current density vs. the square root of the scan rate. (a) and (b) for **1** electrode, (c) and (d) for **2** electrode, (e) and (f) for **4** electrode, (g) and (h) for **5** electrode, respectively.

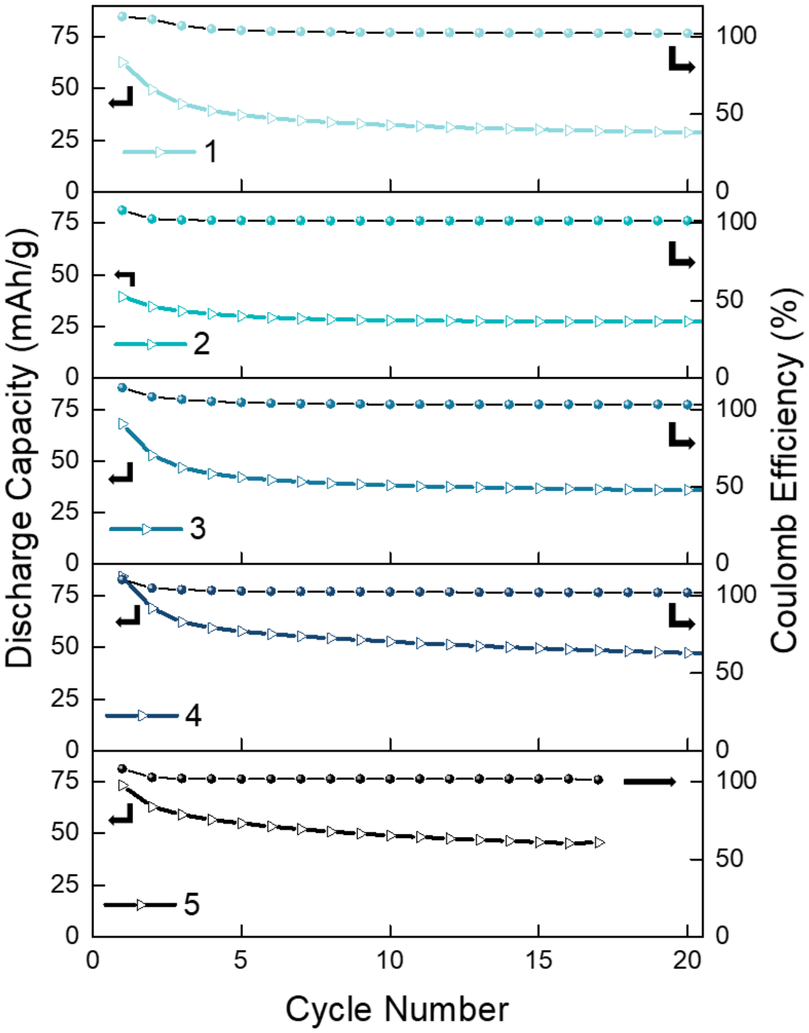

Figure S14. The cycle retention and the corresponding coulomb efficiency of the s-tetrazine electrodes at 0.1 C.

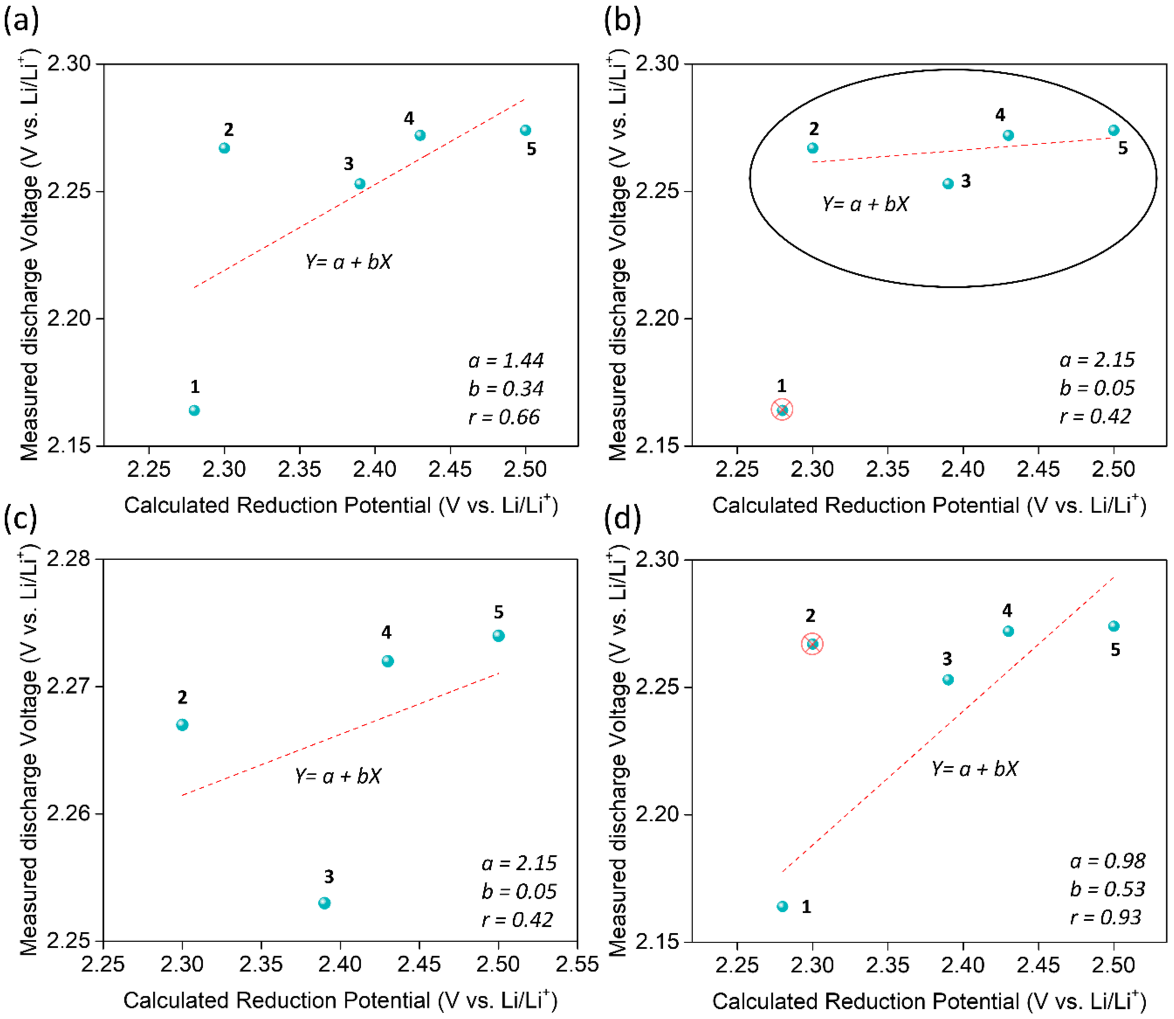

Figure S15. The correlation graphs about the measured discharge voltages in Li-ion cells vs. the calculated reduction potentials considering Li-ion insertion. The dashed red lines indicate linear fitting curves, and the corresponding regression coefficients are presented in the graphs. In the linear regression, all five points were used for (a), but the point 1 and the point 2 were excluded as an outlier for (b) and (d), respectively. The graph (c) is a magnified plot of the circled region in (b).

In the linear regression using all points, it seemed that either the "point 1" or the "point 2" could be an outlier because the both points were far distant from the linearly fitted curve (see Figure S15a). However, when the linear regression was performed without "point 1" as an outlier, the r value was determined to be 0.42 (Figure S15b, c). The low r value indicates lack of linear correlation between the points. Furthermore, the slope b of the fitted line was calculated to be almost zero, implying that the measured discharge voltage was unaffected by the substitution. This is inconsistent to the experimental results that the reduction potential of the s-tetrazines was varied in the CV measurements.

In contrast, when the "point 2" was excluded as an outlier in the linear regression, the r value was determined to be 0.92 (Figure S15d), indicating a strong correlation between the measured discharge voltage and the calculated redox potential. Due to the electron-donating effect of the t-butyl substituents, the 2 electrode was expected to show lower discharge voltage than the 3 electrode. But, in the coin-cell test, only the "point 2" deviated from the calculated trend, which should be most likely attributed not to the electronic effect of the substituents but to other reasons including crystal structure changes during the redox reaction.

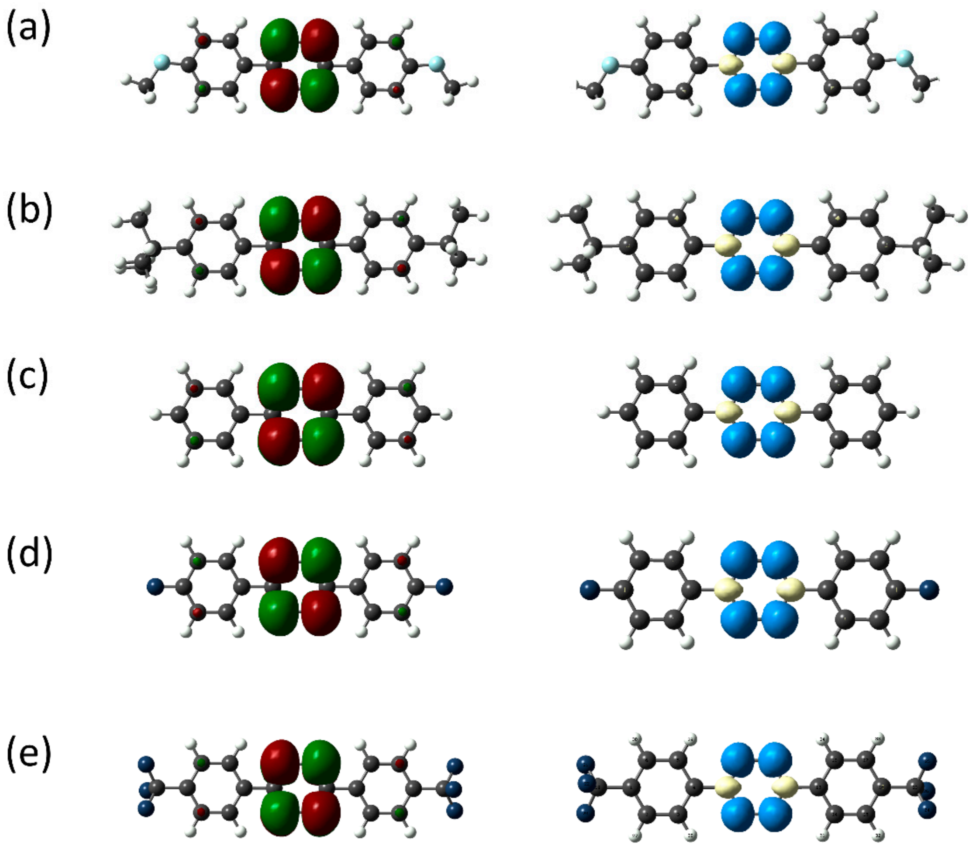

Figure S16. The LUMO orbital of neutral s-tetrazines (left) and the spin density distribution of radical s-tetrazines (right) ((a) **1**, (b) **2**, (c) **3**, (d) **4** and (e) **5**).

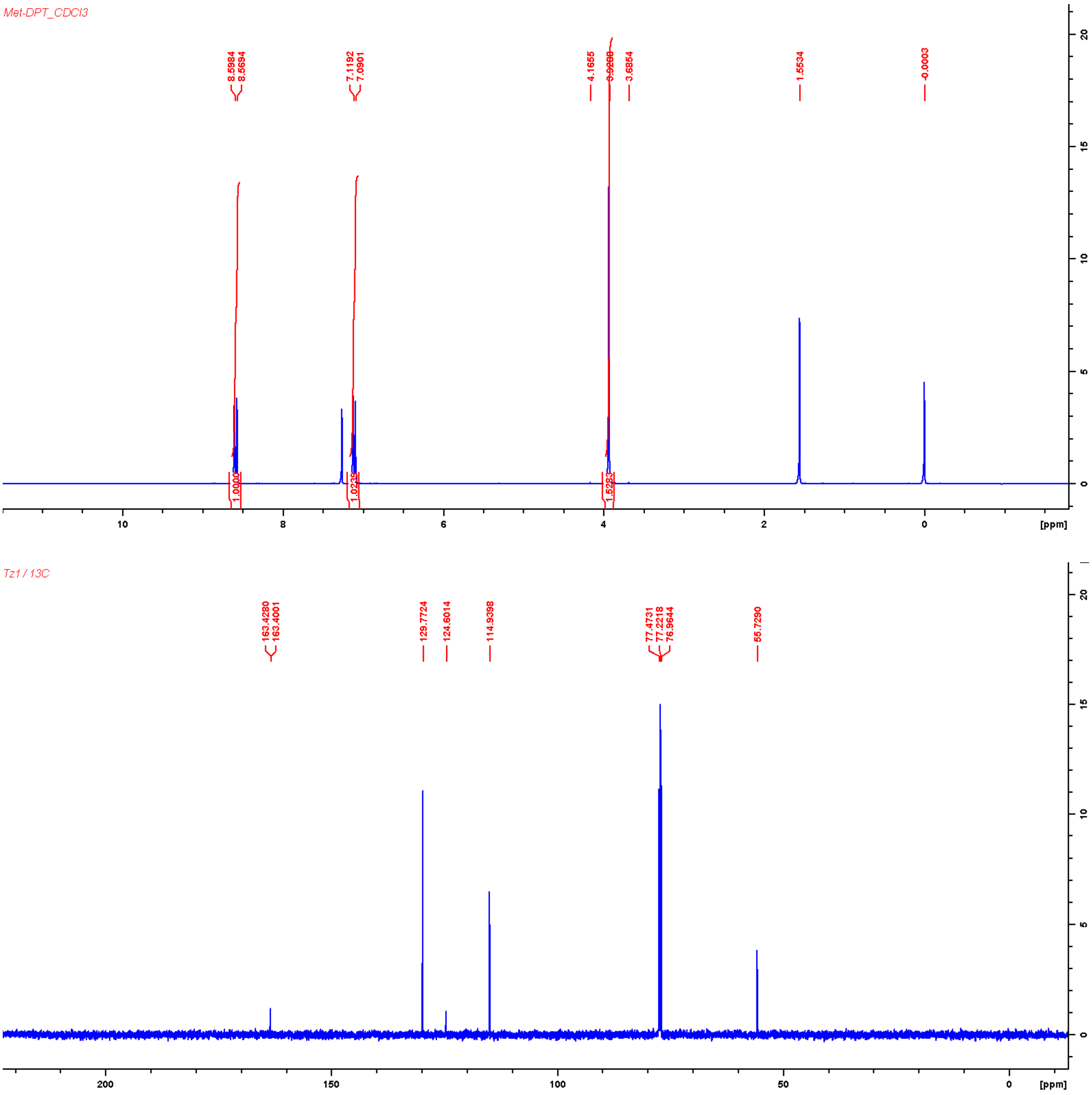

Figure S17. ^1H and ^{13}C NMR spectra of **1**.

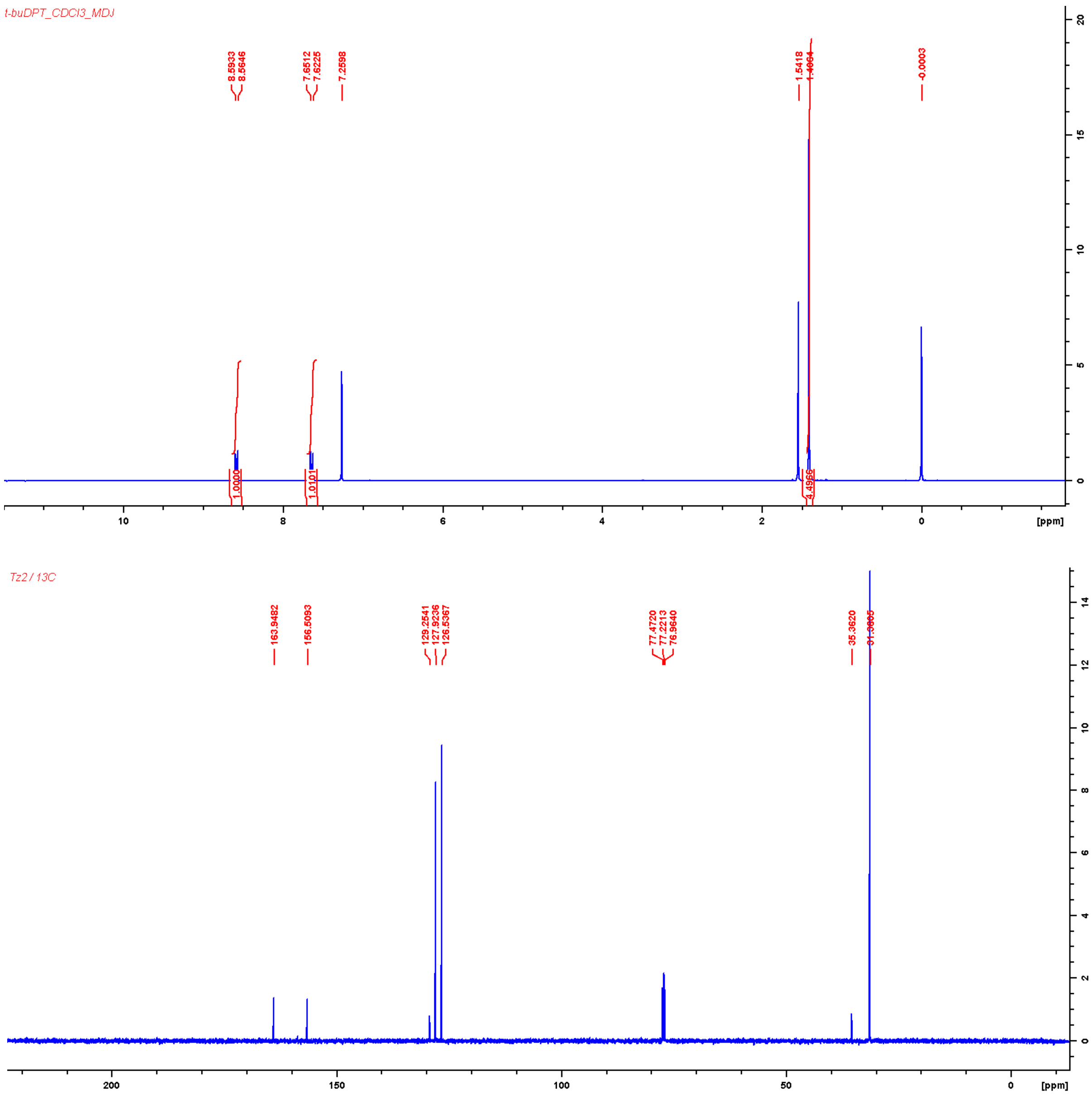

Figure S18. ^{1}H and ^{13}C NMR spectra of **2**.

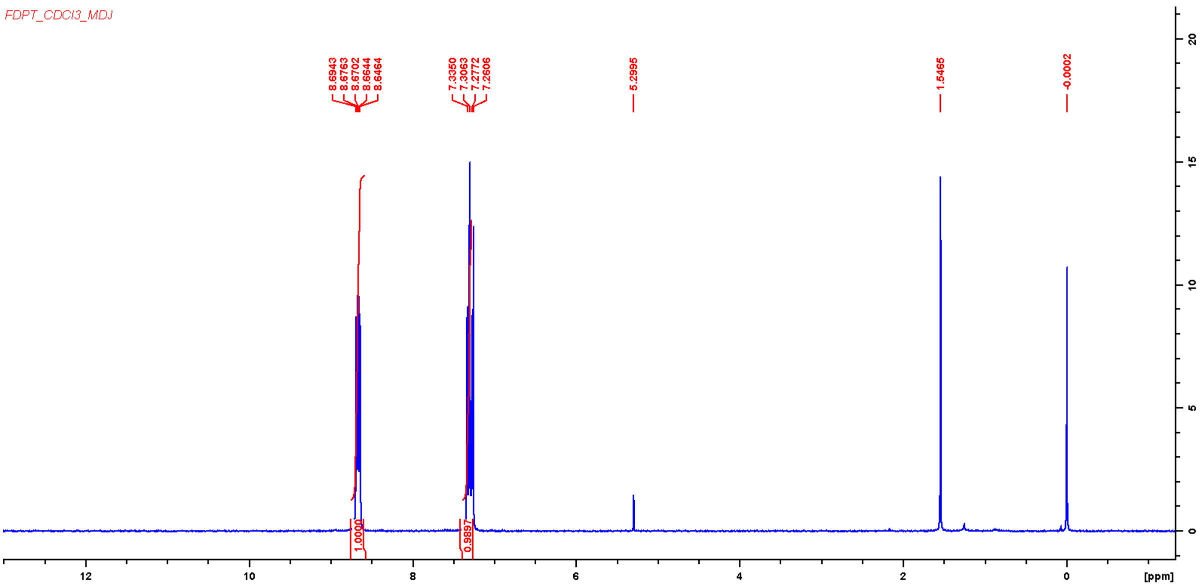

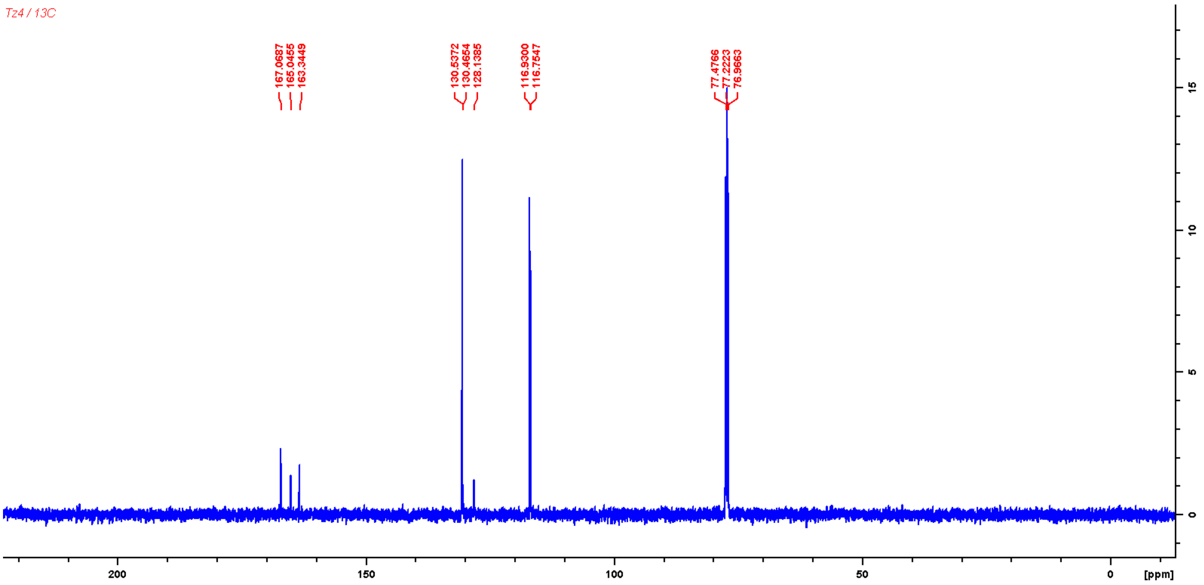

Figure S19. ^{1}H and ^{13}C NMR spectra of **4**.

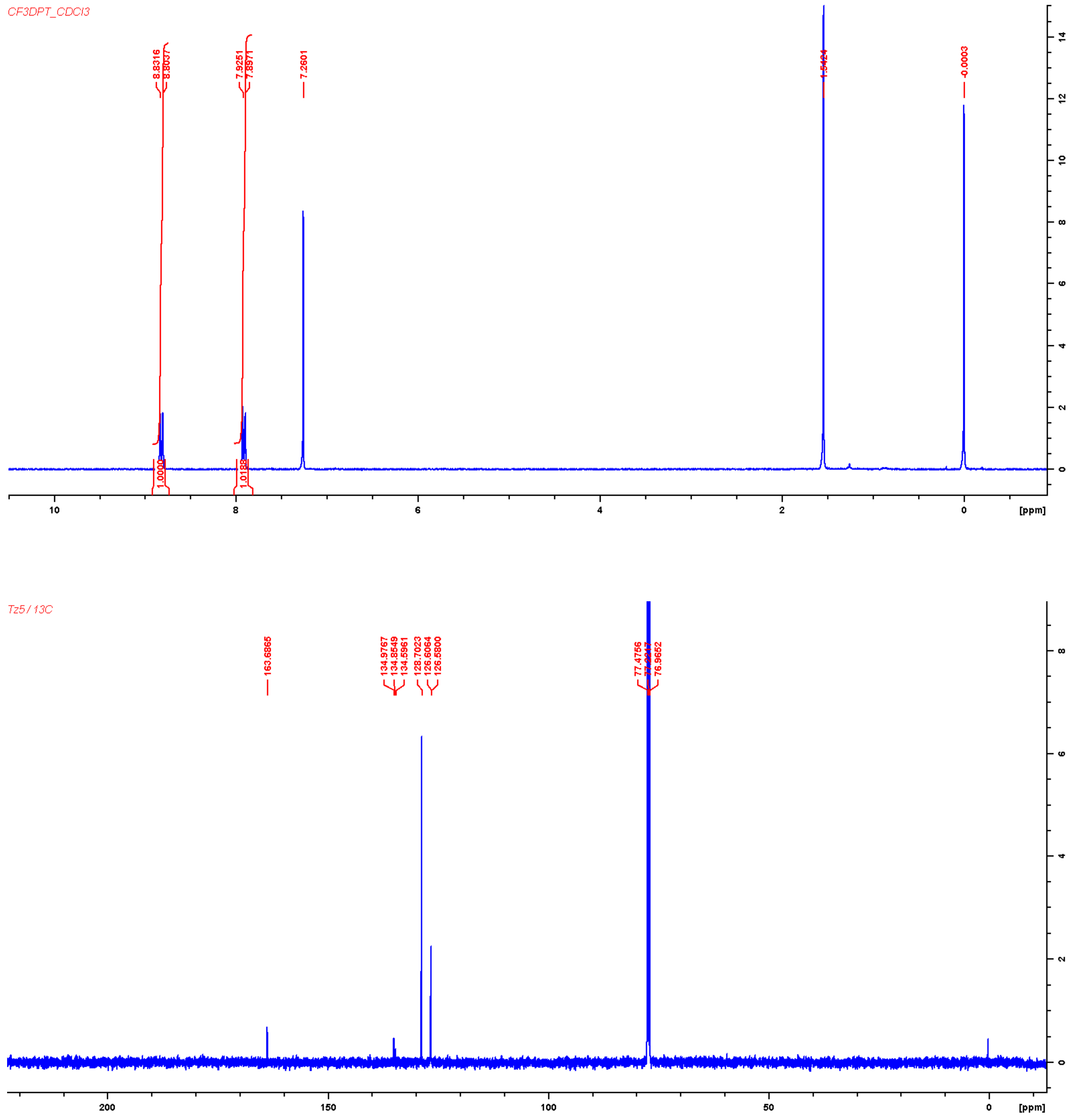

Figure S20. ^{1}H and ^{13}C NMR spectra of **5**.

Table S1. Gibbs Free energies of a Li atom and Li cation.

	Neutral (hartree)	Cation (hartree)	Reduction Potential (V vs. vacuum)
**Li**	-7.517824	-7.447368	1.9172063984

Table S2. Redox potentials of tetrazines without a Li-ion insertion.

	Neutral (hartree)	Anion (hartree)	Reduction Potential (V vs. Li/Li$^+$)
**1**	-987.31591	-987.422041	0.970766695
**2**	-1072.620519	-1072.72875	1.027910635
**3**	-758.313313	-758.42388	1.091476465
**4**	-956.817041	-956.932366	1.220948307
**5**	-1432.437392	-1432.558777	1.385849391

Table S3. Redox potentials of tetrazines with Li cation insertion.

	Neutral (hartree)	Li$^+$ inserted (hartree)	Reduction Potential (V vs. Li/Li$^+$)
**1**	-987.315905	-994.917559	2.280995605
**2**	-1072.620519	-1080.222838	2.299227243
**3**	-758.313327	-765.919175	2.395637233
**4**	-956.817042	-964.424029	2.42627727
**5**	-1432.437392	-1440.046997	2.497489503
